# Pharmacokinetic and pharmacodynamic herb-drug interactions—part I. Herbal medicines of the central nervous system

**DOI:** 10.7717/peerj.16149

**Published:** 2023-11-15

**Authors:** Szilvia Czigle, Milan Nagy, Přemysl Mladěnka, Jaroslav Tóth, the OEMONOM.

**Affiliations:** 1 Department of Pharmacognosy and Botany, Faculty of Pharmacy, Comenius University Bratislava, Bratislava, Slovak Republic; 2 Department of Pharmacology and Toxicology, Faculty of Pharmacy, Charles University, Hradec Králové, Czech Republic

**Keywords:** Herbal medicine-drug interaction, Pharmacokinetic, Pharmacodynamic, *Camellia*, *Valeriana*, *Ginkgo*, *Hypericum*, *Humulus*, *Cannabis*, Review

## Abstract

Unlike conventional drug substances, herbal medicines are composed of a complex of biologically active compounds. Therefore, the potential occurrence of herb-drug interactions is even more probable than for drug-drug interactions. Interactions can occur on both the pharmacokinetic and pharmacodynamic level. Herbal medicines may affect the resulting efficacy of the concomitantly used (synthetic) drugs, mainly on the pharmacokinetic level, by changing their absorption, distribution, metabolism, and excretion. Studies on the pharmacodynamic interactions of herbal medicines and conventional drugs are still very limited. This interaction level is related to the mechanism of action of different plant constituents. Herb-drug interactions can cause changes in drug levels and activities and lead to therapeutic failure and/or side effects (sometimes toxicities, even fatal). This review aims to provide a summary of recent information on the potential drug interactions involving commonly used herbal medicines that affect the central nervous system (*Camellia, Valeriana, Ginkgo, Hypericum, Humulus, Cannabis*) and conventional drugs. The survey databases were used to identify primary scientific publications, case reports, and secondary databases on interactions were used later on as well. Search keywords were based on plant names (botanical genera), officinal herbal drugs, herbal drug preparations, herbal drug extracts.

## Introduction

Medicinal plants and herbal drugs were undoubtedly among the first successful treatment modality ever used by humanity—herbal drugs are currently defined as ‘mainly whole, fragmented or broken plants or parts of plants in an unprocessed state, usually in dried form but sometimes fresh’, (European Pharmacopoeia, 2023h). Even today, the majority of the world’s population still relies on traditional herbal medicines in the broadest sense, and a large part of currently clinically used drugs is of a natural origin or has been derived from it—this fact is also being systematically followed and highlighted, *e.g*., in a continuing series of reviews by Newman and Cragg, at least since 1997 (Newman & Cragg, 2020).

The focus on the issue of herb–drug interactions has been initiated several decades ago by a case report concerning *Citrus* (grapefruit) juice interactions with cardiovascular drugs (that shall be elaborated in the contemporaneously prepared Part II of this review series). Since that, many other herb-drug interaction have been revealed or suggested. Studying herb-drug interactions is complicated by the fact, that there are not only herbal medicines but also herbal food supplements on the world market (N. B.: food supplements—EU terminology, are legally called dietary supplements in the USA) (Czigle et al., 2018). For many popular medicinal plant extracts, the same particular (standardized) extract can be found in medicinal and food products at the same time on the same local market (Czigle et al., 2018). Even that food products should be marketed with lower doses of these extracts, as they must not be intended to treat human diseases. Regardless, even if the ingredients of supplements are labelled as part of the traditional human diet by manufacturers, and their effects claimed physiological, *i.e*., maintaining human health, such herbal materials (herbal drugs, botanicals, and extracts) certainly contain the same biologically active constituents, no matter if they are part of medicinal or food products. These active compounds can interact with other medications, herbal or synthetic, at different levels (Cordier & Steenkamp, 2011; Singh & Zhao, 2017).

In contrast to well-investigated novel drugs which must undergo long-term preclinical and clinical investigations with the aim of defining indications, mechanism of action, as well as their undesirable effects, contraindications, and possible interactions, many phytopharmaceuticals profit from their traditional status (European Medicines Agency, 2023). This means that detailed information including data from clinical studies is mostly missing. A quite dangerous subconscious assumption has evolved, that all what comes from nature is safe (Ng, 2021). The need for new data on herbal efficacy and safety has been highlighted by recent systematic regulatory approaches in the herbal medicines segment, most prominently represented by the works of the European Medicines Agency—Committee on Herbal Medicinal Products (EMA-HMPC). European Union herbal monographs typically also contain chapters on contraindications, undesirable effects, interactions with other medicinal products, warnings, *etc*. (European Medicines Agency, 2023). Contrary to indications and mechanisms of action, information on less frequent undesirable effects and interactions with other medicinal products is often gained in the clinical trials phase IV, *i.e*., in post-market surveillance studies and/or from less systematic and less predictable sources, *e.g*., individual case reports. The latter usually comes from a casual overdose incident. The prevalence of use of particular herbal medicines in the general population is so well reflected in the number of clinically important herb-drug interactions known and described so far for these herbal sources (Prely et al., 2022; TRC, 2023; Williamson, Driver & Baxter, 2009, 2018; Zhang et al., 2022; Ziemann et al., 2019).

Based on recently published information, this work aims to sum up the knowledge about interactions between conventional drugs and (traditional) herbal drugs that affect the central nervous system (psychopharmaceuticals) at the level of vigilance, affectivity, and psychical integrity. Vigilance is affected by psychostimulants—herbal caffeine sources (guarana—Guaranae semen/Paulliniae semen, green/black tea—Camelliae folium, cola—Colae semen, coffee—Coffeae semen, maté leaf—Mate folium, cocoa—Cacao semen), hypnotics (valerian root—Valerianae radix) and nootropics (*Ginkgo* leaf—Ginkgonis folium). The affectivity is impacted by antidepressants (St. John’s wort—Hyperici herba) and anxiolytics (hop strobile—Lupuli flos), and psychical integrity by hallucinogens (cannabis aerial part/marijuana—Cannabis herba, cannabis resin/hashish—Cannabis resina).

A drug interaction is said to occur when the effect of one drug is changed by the presence of another substance, including herbal medicines, foods (supplements), beverages, and environmental agents. The outcome can be harmful if the interaction causes an increase in the side effect and/or toxicity of the drug (Cordier & Steenkamp, 2011; Singh & Zhao, 2017). A reduction in efficacy due to an interaction can sometimes be just as harmful as an increase. Patients can apparently tolerate adverse interactions remarkably well and many interactions can be accommodated, so effects may not be consciously recognized as the result of an interaction. The mechanism of herbal interaction might not be easily tracked, as herbal sources differ from conventional drug substances in that they are complex mixtures of many biologically active compounds (Nagy, Mučaji & Grančai, 2017). The resulting pharmacological action is likely to arise often on the basis of synergism. Explaining so-called herb-drug interactions is therefore often more difficult. Furthermore, many drugs can interact by more than a single mechanism. Sometimes, more mechanisms are acting in concert. For clarity, most of the mechanisms that are dealt with in this review are as though they occur in isolation (Nagy, Mučaji & Grančai, 2017). For convenience, the mechanism of interactions can be divided into those that involve the pharmacokinetics of a drug, and those that are pharmacodynamic (Cordier & Steenkamp, 2011; Singh & Zhao, 2017; Williamson, Driver & Baxter, 2009).

Pharmacokinetic interactions are those that can affect the processes by which drugs are absorbed, distributed, metabolized, and eliminated (the so-called ADME interactions) (Cordier & Steenkamp, 2011; Singh & Zhao, 2017; Williamson, Driver & Baxter, 2009, 2013). The classical example of a target is the most well-investigated drug transporter efflux transporter P-glycoprotein (P-gp), also known as multidrug resistance protein 1 (MDR1) or ATP-binding cassette sub-family B member 1 (ABCB1) (Williamson, Driver & Baxter, 2009). Some well-known examples include ginkgo (maidenhair tree)—*Ginkgo biloba* (with active ginkgolides) is an inhibitor of P-gp and St. John’s wort—*Hypericum perforatum* is an inductor of P-gp (Czigle & Tóth, 2016). Other transporters that are involved in some drug interactions are organic cation transporters (OCTs) and organic anion-transporting polypeptides (OATPs) (Ali et al., 2020; Rodrigues et al., 2019). Although all of these mechanisms are undoubtedly relevant for interactions with herbal medicines, this review will focus mainly on cytochrome P_450_ and the P-gp drug transporter protein, as information on other transporters is currently limited or of unknown biological relevance. Some of the secondary metabolites are enzyme inductors or inhibitors (CYP isoenzymes) (Amaeze et al., 2021; Manikandan & Nagini, 2018; Pandian et al., 2020; Wanwimolruk & Prachayasittikul, 2014; Williamson, Driver & Baxter, 2009; Zhou et al., 2003). With the exception of prodrugs, inhibitors slow down drug metabolism, which often leads to a substantial increase in serum drug levels with significant risk of adverse or even toxic effects (Cordier & Steenkamp, 2011; Singh & Zhao, 2017). Inductors cause the opposite, increased drug metabolism. Again, with the exception of prodrugs, this consequently often leads to lower-than-expected serum drug levels with potential treatment failure (Williamson, Driver & Baxter, 2009). St. John’s wort—*Hypericum perforatum* is a well-known inductor of CYP isoenzymes (Czigle & Tóth, 2016; Williamson, Driver & Baxter, 2009), inhibitors include ginkgo—*Ginkgo biloba* (Czigle & Tóth, 2016; Williamson, Driver & Baxter, 2009), valerian—*Valeriana officinalis* (Czigle & Tóth, 2016; Williamson, Driver & Baxter, 2009), hop—*Humulus lupulus* (Czigle & Tóth, 2016; Williamson, Driver & Baxter, 2009), among others (Czigle & Tóth, 2016; Williamson, Driver & Baxter, 2009). Finally, several other enzymes have been shown to play a role in herb-drug interactions, such as UDP-glucuronyltransferases (UGT) (Williamson, Driver & Baxter, 2009, 2018).

Pharmacodynamic interactions are those where the effects of one drug are changed by the presence of another drug at its site of action. Sometimes the drugs and the biologically active plant metabolites directly compete for a particular target (*e.g*., receptor, enzyme), but often the reaction is more indirect and involves interference with physiological mechanisms. If two drugs that have the same or similar pharmacological effect are given together, the effects can be additive and, in some cases, even potentiating (Nagy, Mučaji & Grančai, 2017). In contrast, there are some pairs of drugs with activities that are opposed to each other (antagonist) (Nagy, Mučaji & Grančai, 2017). For example, a particular additive effect is well known to occur between ginkgo—*Ginkgo biloba* and antithrombotic agents (Czigle & Tóth, 2016; European Medicines Agency, 2012d) and similarly between St. John’s wort—*Hypericum perforatum* and antidepressant drugs (Czigle & Tóth, 2016; European Medicines Agency, 2021). The exposures to drugs, for which knowledge of the potential adverse herb-drug interactions is lacking, might be a risk to patients’ safety (Czigle & Tóth, 2016; Williamson, Driver & Baxter, 2009, 2018). On the contrary, some interactions may be therapeutically beneficial (Czigle & Tóth, 2016; Williamson, Driver & Baxter, 2009, 2018) and might be used to develop new therapeutic strategies in the future.

## Survey methodology

This work aims to summarize the knowledge about interactions between conventional drugs and (traditional) herbal drugs; and, as part of an intended series on this topic, concentrates on principal herbal drugs that affect psychopharmaceuticals. Subheads include herbal drugs that affect the central nervous system at the level of vigilance, affectivity, and psychical integrity. The given topic has been comprehensively summarized from the current scientific view on herbal medicines and has been currently (re-)evaluated by the Evidence Based Medicine approach (factor—risk/benefit ratio; the beneficial effects of an herbal medicine can outweigh the side effect). The minimum acceptance criteria were similar to the guidelines for authors of reviews by Munn et al. (2018). The survey databases used were SciFinder, PubMed, which identified primary scientific publications (*e.g*., case reports), and several secondary databases on interactions were used later on as well (Therapeutic Research Center—Natural Medicines database (TRC, 2023), Stockley’s Herbal Medicines Interactions (Williamson, Driver & Baxter, 2009, 2013, 2018), and other databases). The search was focused on officinal herbal drugs (European Pharmacopoeia, 2023h), herbal drug preparations (European Pharmacopoeia, 2023g), and herbal drug extracts (European Pharmacopoeia, 2023f). Keywords for search are listed here: herbal medicine-drug interaction, pharmacokinetic and pharmacodynamic interaction, selected botanical genera: *Camellia*, *Coffea*, *Paullinia*, *Ilex*, *Cola, Theobroma*, *Valeriana*, *Ginkgo*, *Hypericum*, *Humulus*, *Cannabis*. Subsequently, the names of the particular herbal drugs were included in the search, as Camelliae folium non fermentatum/fermentatum, Coffeae semen, Guaranae semen, Mate folium, Colae semen, Cacao semen, Valerianae radix, Ginkgonis folium, Hyperici herba, Humuli flos, Cannabis flos/herba/resina. Some botanical synonyms and related terms. We compiled information about pharmacokinetic and pharmacodynamic interactions with biologically active metabolites of these plants or herbal drugs. The criteria for the inclusion or exclusion of specific literature sources were interaction results, the conclusions of case report evaluations, clinical relevance of particular interactions or their absence, and the exhaustiveness of the description of the interaction. The same herbal medicines are often used in multiple indications, in different individual diagnoses; on the other hand, the mechanism of action of particular secondary metabolites is related to several diagnoses by EU herbal monographs (European Medicines Agency, 2023). Our objective was to explain the biological mechanisms of pharmacological activities for particular secondary metabolites in individual indications. The resulting pharmacological effect often is due to the synergism of the individual secondary metabolites. As for legislative aspects, the European Medicines Agency (EMA) applies the same criteria on all herbal drugs and on all diseases/health problems in its given methodological procedures. We summarize, synthesize, paraphrase data, and give an overview of the main points out of each source and combine them into a coherent whole. We analyze and interpret (clinically relevant) herb-drug interactions in the tables in the next sections with clarification, notes after analyses of theoretical or practical information. The Anatomical Therapeutic Chemical (ATC) classification system of the World Health Organization (WHO) Collaborating Centre for Drug Statistics Methodology (World Health Organization, 2023) is used to order and organize drug molecules in tables to highlight links in therapeutic and pharmacological/chemical subgroups. Further criteria reflected in this article include the availability of information about the potential of interaction of the particular drug, about interactions prevention measures, as well as practical recommendations after the manifestation of the interaction.

## Drug interactions of herbal drugs containing caffeine (Guaranae semen, Camelliae folium, Colae semen, Coffeae semen, Mate folium, Cacao semen)

The most common herbal drugs used as sources of caffeine include green and black tea—Camelliae folium non fermentatum/fermentatum (Theae folium), coffee—Coffeae semen, guarana—Guaranae semen (Paulliniae semen, Guarana, Pasta guarana), maté leaf—Mate folium, cola—Colae semen, and cocoa—Cacao semen. Significantly, in addition to their use in herbal medicinal products, all of these herbal sources are commonly present in foods and beverages, as well (tea, coffee, cola drinks, and energy drinks) (Czigle & Tóth, 2010b). The caffeine content in the named herbal drugs decreases in the following order: Guarana (3.6–6%) > Camelliae folium (2–4%) > Colae semen (1–2.5%) > Coffeae semen (1–2%) > Mate folium (0.5–0.8%) > Cacao semen (0.07–0.4%) (Czigle & Tóth, 2010b). Camelliae folium and Colae semen additionally contain appreciable amounts of tannins, for Colae semen and Coffeae semen the presence of chlorogenic acids, and for Cacao semen the content of fatty acids is substantial (Czigle & Tóth, 2010b; Nagy, Mučaji & Grančai, 2017).

The European Pharmacopoeia lists officinal monographs for: green tea (Camelliae sinensis non fermentata folia) (European Pharmacopoeia, 2023d), guarana (Guaranae semen) (European Pharmacopoeia, 2023e), cola (Colae semen) (European Pharmacopoeia, 2023a), and maté leaf (Mate folium) (European Pharmacopoeia, 2023j). Coffee (Coffeae semen, *Coffea* L. spp., Rubiaceae) and cocoa (Cacao semen, *Theobroma cacao* L., Malvaceae) are not officinal in the European Pharmacopoeia.

The plant species *Camellia sinensis* (L.) Kuntze (Theaceae), with the botanical synonym *Thea sinensis* L., is known as the source of green tea (Camelliae folium non fermentatum), black tea (Camelliae folium fermentatum) and oolong (Camelliae folium semifermentatum) (The World Flora Online, 2022). There is a wide array of indications, well supported by in-depth studies on a number of defined chemical constituents of *Camellia sinensis* (L.) Kuntze. The main biologically active compounds are xanthine derivatives, such as caffeine (2–4%), theobromine (0.05%) and theophylline (traces); polyphenols (up to 30%), including gallotannins, (-)-epigallocatechin-3-*O*-gallate (EGCG, up to 12%), (-)-epicatechin-3-*O*-gallate (ECG), catechin tannins, other flavonoids, ellagitannins, phenolic carboxylic acids; theanine (amino acid), ascorbic acid, triterpene saponins, and minerals (Nagy, Mučaji & Grančai, 2017).

The dried seed of *Paullinia cupana* Kunth (syn. *Paullinia sorbilis* Mart.) (Sapindaceae), guarana, is also known as a food product, often available in the form of candy lozenges with Pasta guarana (guarana extract) (Czigle & Tóth, 2010b).

The dried leaf of *Ilex paraguariensis* A.St.-Hil. (Aquifoliaceae) is known as maté tea. After harvesting, the leaf is rapidly desiccated by heating, and cut (Czigle & Tóth, 2016).

Cola is produced from the plant species *Cola nitida* (Vent.) Schott et Endl. (syn. *C. vera* K. Schum.) and its varieties, as well as from *Cola acuminata* (P. Beauv.) Schott et Endl. (syn. *Sterculia acuminata* P. Beauv.) (Malvaceae); the herbal drug consists of whole or fragmented dried seeds, which were freed from the testa. Cola drinks contain extracts of this herbal drug (Czigle & Tóth, 2016).

Caffeine-containing herbal drugs with psychostimulant effects are used medicinally ‘for the relief of symptoms of (temporary) fatigue and sensation of weakness’, (European Medicines Agency, 2023). Based on a large amount of verified scientific data, several of these herbal drugs have been classified as traditional herbal medicinal products with the above cited indication: Camelliae folium non fermentatum (whole dried leaf, comminuted and powdered herbal substance) (European Medicines Agency, 2012c), Guaranae semen (guarana seed in solid dosage forms for oral use) (European Medicines Agency, 2011b), Colae semen (as a powdered herbal substance in solid dosage forms or as a herbal tea for oral use, as liquid extract or tincture) (European Medicines Agency, 2010b), Mate folium (as a comminuted herbal substance such as herbal tea for oral use) (European Medicines Agency, 2008b). In addition to this general therapeutic indication, maté leaf was also classified as a traditional herbal medicinal product ‘to increase the amount of urine to achieve flushing of the urinary tract as an adjuvant in minor urinary complaints’, (European Medicines Agency, 2008b).

Caffeine itself is a CYP1A2 inhibitor (Guo et al., 2021; Kot & Daniel, 2008; Williamson, Driver & Baxter, 2013). Caffeine metabolites are paraxanthine (84%), theobromine (12%), and theophylline (4%) (Faber, Jetter & Fuhr, 2005). In commonly achievable plasma concentrations, caffeine is an antagonist at adenosine A_1_ and A_2_-receptors (Cauli & Morelli, 2005; Nehlig, 2018). In higher concentrations, it acts as well as a phosphodiesterase inhibitor (PDE1, PDE4, PDE5) (Boswell-Smith, Spina & Page, 2006); γ-aminobutyric acid receptor (GABA_A_) antagonist (Isokawa, 2016; Lopez et al., 1989); ryanodine receptor (RYR) agonist (Kong et al., 2008); and it also affects the *nervus vagus* (Hibino et al., 1997). Caffeine and paraxanthine have similar sympathomimetic actions (Benowitz et al., 1995).

This review presents and summarizes relevant and more recent data on the interaction potential of caffeine and caffeine-containing herbal drugs that are, notably, not included in the European Union herbal monographs and their related assessment reports (European Medicines Agency, 2008a, 2010a, 2011a, 2012a, 2020a, 2022).

Clinically significant pharmacokinetic and pharmacodynamic interactions are summarized in Table 1. In relation of pharmacodynamic interactions, it is important to emphasize that preparations containing caffeine reduce the sedative action and increase the side effects caused by sympathomimetic drugs (European Medicines Agency, 2008b, 2010b, 2011b, 2012c). Patients taking monoamine oxidase (MAO) inhibitors should be cautious when concomitantly using Paulliniae semen (European Medicines Agency, 2011b), Mate folium (European Medicines Agency, 2008b), and/or Colae semen (European Medicines Agency, 2010b). However, there are, also some important pharmacokinetic interactions based either on the inhibition of CYP1A2 metabolism of other drugs by caffeine (*e.g*., melatonin) (European Medicines Agency, 2008b, 2010b, 2011b, 2012c) or by inhibition of the same cytochrome by other drugs resulting in potential caffein toxicity (*e.g*., fluvoxamine, ciprofloxacine) (European Medicines Agency, 2008b, 2010b, 2011b, 2012c).

**Table 1:** Drug interactions of caffeine and herbal drugs containing caffeine.

ATC therapeutic subgroup	ATC pharmacological/chemical subgroup	Drugs	Pharmacokinetic basis of interaction	Pharmacodynamic basis of interaction	Manifestation of interaction	
Stomatological preparations (A01)	Vasoconstrictors	Epinephrine (Adrenaline)	CYP1A2	Caffeine is a competitive adenosine antagonist and releases endogenous catecholamines.	additive (psychostimulant) effect **↑** adverse reactions (hypertension, myocardial infarction, stroke, seizures) **!** reduce/restrict caffeine intake	Van Soeren et al. (1996)
			CYP3A4			
Antihemorrhagics (B02)	Local hemostatics					
Cardiac therapy (C01)	Adrenergic agents					
Nasal preparations (R01)	Decongestants and other nasal preparations for topical use Sympathomimetics, plain					
Drugs for obstructive airway diseases (R03)	Adrenergic inhalants, alpha- and beta-adrenoreceptor agonists					
Ophtalmologicals (S01)	Sympathomimetics in glaucoma therapy					
Stomatological preparation, in dentistry (A01)	Salicylic acid and derivatives	Acetylsalicylic acid	CYP1A2 CYP2C9 CYP3A4		**↑** bioavailability **↑** analgetic effect	Diener et al. (2005)
Antithrombotic agents (B01)						
Analgetics (N02)						
Drugs for acid related disorders (A02)	H_2_-receptor antagonists	Cimetidine	CYP2C9		**↑** caffeine plasma level **↓** caffeine clearance (31–42%) **↑** caffeine adverse reactions **!** reduce/restrict caffeine intake	Broughton & Rogers (1981)
	Proton pump inhibitors	Omeprazol	CYP2C9		**↑** caffeine plasma level **↓** caffeine clearance **↑** caffeine adverse reactions **!** reduce/restrict caffeine intake	Andersson et al. (1998)
Antiobesity preparations, excl. diet products (A08)	Centrally acting antiobesity products	Amfepramone Phentermine	CYP1A2 CYP3A4	Caffeine is a competitive adenosine antagonist and releases endogenous catecholamines.	additive (psychostimulant) effect **↑** adverse reactions (hypertension, myocardial infarction, stroke, seizures) **!** reduce/restrict caffeine intake	Solimini et al. (2017)
Antiobesity preparations, excl. diet products (A08)	Centrally acting antiobesity products	Ephedrine (orally)	CYP1A2 CYP3A4	Caffeine is a competitive adenosine antagonist and releases endogenous catecholamines.	additive effect **!** life-threatening risk (hypertension, myocardial infarction, stroke, seizures, even death) **!** reduce/restrict caffeine intake	Astrup et al. (1992)
Cardiac therapy (C01)	Adrenergic and dopaminergic agents					
Nasal preparations (R01)	Decongestants and other nasal preparations for topical use; sympathomimetics, plain Sympathomimetics, combinations excl. corticosteroids					
Drugs for obstructive airway diseases (R03)	Alpha- and beta-adrenoreceptor agonists					
Ophthalmologicals (S01)	Sympathomimetics excl. antiglaucoma preparations					
Drugs used in diabetes (A10)	Blood glucose lowering drugs, excl. insuline, sulfonylureas	Glibornuride Glimepiride	CYP2C9	Glucose tolerance affected.	glucose level affected **!** monitor glucose level(s)	Czigle & Tóth (2016), Williamson, Driver & Baxter (2018)
	Blood glucose lowering drugs, excl. insuline, thiazolidinedione	Pioglitazone Rosiglitazone	CYP2C9	Glucose tolerance affected.	glucose level affected **!** monitor glucose level(s)	Czigle & Tóth (2016), Williamson, Driver & Baxter (2018)
Antithrombotic agents (B01)	Platelet aggregation inhibitors excl. heparin	Clopidogrel Dipyridamole Ticlopidine	CYP1A2 CYP2D6	Caffeine is a competitive adenosine antagonist.	**↑** bioavailability **↑** antithrombotic effect **!** bleeding risk **!** do not use combination (24 h interval)	Birnbaum et al. (2015)
	Vitamin K antagonists	Warfarin	CYP1A2 CYP2C9		**↑** anticoagulant effect **!** bleeding risk **!** reduce/restrict caffeine intake	Anthony et al. (2009)
	Heparin group	Ardeparin Dalteparin Enoxaparin Heparin	CYP1A2 CYP2C9		**↑** anticoagulant effect **!** bleeding risk **!** reduce/restrict caffeine intake Ardeparin was recalled from circulation for reasons unrelated to safety or efficacy	Anthony et al. (2009)
Cardiac therapy (C01)	Antiarrhythmics, class Ib	Lidocaine Mexiletine Tocainide	CYP1A2 CYP2D6	Caffeine is a competitive adenosine antagonist.	**↑** caffeine plasma level **↑** caffeine adverse reactions **!** reduce/restrict caffeine intake	Amjad et al. (2017), Czigle & Tóth (2016)
	Antiarrhythmics, class Ic	Flecainide	CYP2D6	Caffeine is a competitive adenosine antagonist.	**↑** caffeine plasma level **↑** caffeine adverse reactions **!** reduce/restrict caffeine intake	Hwang et al. (2019)
Antifungals for dermatological use (D01)	Antifungals for systemic use/allylamine antifungals	Terbinafine	CYP1A2 CYP3A4		**↑** caffeine plasma level **↓** caffeine clearance (19%) **↑** caffeine effect prolongation **!** reduce/restrict caffeine intake	Trépanier, Nafziger & Amsden (1998)
Sex hormones and modulators of the genital system (G03)	Hormonal contraceptives for systemic use	Ethinylestradiol (EE), Estradiol + progestogens	CYP1A2 CYP2C9 CYP3A4	Estrogens are inhibitors of caffeine metabolism.	**↑** caffeine plasma level **↓** caffeine clearance (40–65%) **!** reduce/restrict caffeine intake	Fantoli (1981), Patwardhan et al. (1980)
Antibacterials for systemic use (J01)	Quinolone antibacterials	Ciprofloxacin Clinafloxacin Enoxacin Gatifloxacin Grepafloxacin Levofloxacin Lomefloxacin Moxifloxacin Norfloxacin Ofloxacin Pipemidic acid Rufloxacin Sparfloxacin Trovafloxacin	CYP1A2 CYP3A4		**↑** caffeine plasma level **↓** caffeine metabolism (*N*-demethylation) **↓** caffeine clearance **↑** adverse reactions **!** reduce/restrict caffeine intake	Czigle & Tóth (2010b), Woziwodzka et al. (2022)
Antimycotics for systemic use (J02)	Triazole and tetrazole derivatives—antimycotics	Fluconazol	CYP1A2 CYP3A4		**↓** caffeine clearance (25%) **!** reduce/restrict caffeine intake	Czigle & Tóth (2010b)
Drugs for treatment of bone diseases (M05)	Drugs affecting bone structure and mineralization, bisphosphonates	Alendronic acid	CYP1A2 CYP3A4		**↓** bioavailability **!** reduce/restrict caffeine intake (2 h interval)	Czigle & Tóth (2010b)
Analgesics (N02)	Other analgesics and antipyretics, anilides	Paracetamol	CYP1A2 CYP3A4 CYP2E1		**↑** bioavailability **↑** analgetic effect	Diener et al. (2005), Granados-Soto & Castañeda-Hernández (1999), Zhang (2001)
	Antimigraine preparations, ergot alkaloids	Ergotamine	CYP1A2 CYP3A4	Caffeine vigilance increased.	**↑** absorbance **!** reduce/restrict caffeine intake	Granados-Soto & Castañeda-Hernández (1999)
Antiepileptics (N03)	Antiepileptics, barbiturates and derivatives	Phenobarbital	CYP2C9	Caffeine vigilance increased.	**↓** plasma level **↓** anticonvulsive effect **!** reduce/restrict caffeine intake	Chrościńska-Krawczyk et al. (2014), Jankiewicz et al. (2007)
	Antiepileptics, hydantoin derivatives	Phenytoin	CYP1A2 CYP3A4	Caffeine vigilance increased.	**↓** plasma level **↓** anticonvulsive effect **!** reduce/restrict caffeine intake	Chrościńska-Krawczyk et al. (2014), Nomani et al. (2019)
	Antiepileptics, benzodiazepine derivatives	Clonazepam	CYP1A2 CYP3A4	Caffeine vigilance increased.	**↓** plasma level **↓** anticonvulsive effect **!** reduce/restrict caffeine intake	Chrościńska-Krawczyk et al. (2014), Nomani et al. (2019)
	Antiepileptics, carboxamide derivatives	Carbamazepine	CYP3A4 CYP2C8	Caffeine vigilance increased.	**↓** plasma level **↓** anticonvulsive effect **!** reduce/restrict caffeine intake	Chrościńska-Krawczyk et al. (2014), Jankiewicz et al. (2007), Nomani et al. (2019)
	Antiepileptics, fatty acid derivatives	Valproic acid	CYP2C9 CYP2A6	Caffeine vigilance increased.	**↓** plasma level **↓** anticonvulsive effect **!** reduce/restrict caffeine intake	Chrościńska-Krawczyk et al. (2014), Nomani et al. (2019)
Psycholeptics (N05)	Antipsychotics	Clozapine Fluphenazine Haloperidol Chlorpromazine Lithium Prochlorperazine Thioridazine Triflupromazine	CYP1A2 CYP2D6	Caffeine vigilance increased.	**↑** clozapine plasma level **↑** clozapine toxicity (400–1,000 mg caffeine per day) **↓** lithium plasma level (tremor possible after abrupt caffeine withdrawal) **!** exacerbation of psychotic symptoms (possible) **!** reduce/restrict caffeine intake **!** patient monitoring necessary	Carrillo & Benitez (2000)
	Anxiolytics, benzodiazepine derivatives	Alprazolam Diazepam	CYP1A2 CYP3A4	Caffeine vigilance increased.	**↓** plasma level **↓** anxiolytic effect **!** reduce/restrict caffeine intake	Loke, Hinrichs & Ghoneim (1985), Roache & Griffiths (1987)
	Hypnotics and sedatives	Pentobarbital Triazolam Zolpidem Zopiclon	CYP2C2	Caffeine vigilance increased.	**↓** hypnotic effect **!** reduce/restrict caffeine intake	Batéjat et al. (2006), Walsh, Muehlbach & Schweitzer (1995)
	Melatonin receptor agonists	Melatonin				Harpsøe et al. (2015), Härtter et al. (2006)
Psychoanaleptics (N06)	Antidepressants, non-selective monoamine reuptake inhibitors	Amitriptyline Imipramine	CYP1A2 CYP2C19	Caffeine vigilance increased.	**↑** caffeine plasma level **↑** adverse reactions **!** reduce/restrict caffeine intake	Sawynok (2011)
	Antidepressants, selective serotonin reuptake inhibitors	Fluvoxamine	CYP1A2 CYP2C19	Caffeine vigilance increased.	**↑** caffeine plasma level **↑** adverse reactions **!** reduce/restrict caffeine intake	Carrillo & Benitez (2000)
Other nervous system drugs (N07)	Drugs used in nicotine dependence	Nicotine (transdermally)	CYP2C9		**↑** of caffeine plasma level **↓** caffeine clearance **!** exclude caffeine (intoxication risk)	Kroon (2007)
	Drugs used in alcohol dependence	Disulfiram	CYP1A2 CYP3A4		**↑** caffeine plasma level **↓** caffeine clearance **!** exclude caffeine (intoxication risk)	Beach et al. (1986)
	Other nervous system drugs, multiple sclerosis treatment	Riluzole	CYP1A2	Caffeine vigilance increased.	**↑** caffeine and riluzole plasma level **!** reduce/restrict caffeine intake	van Kan et al. (2005)
Nasal preparations (R01)	Sympathomimetics	Phenylpropanolamine Pseudoephedrine	CYP1A2	Caffeine vigilance increased.	**↑** caffeine plasma level **↑** adverse reaction (hypertension) **!** dosage adjustment	Jones (2008), Pentel (1984)
Drugs for obstructive airway diseases (R03)	Non-selective beta-adrenoreceptor agonists)	Isoprenaline (Isoproterenol) Orciprenaline (Metaproterenol)	CYP1A2		**↑** positive inotropic effect of β-sympathomimetics **!** reduce/restrict caffeine intake	Czigle & Tóth (2010b)
	Selective beta-2-adrenoreceptor agonists	Salbutamol (Albuterol) Terbutaline	CYP1A2		**↑** positive inotropic effect of β-sympathomimetics **!** reduce/restrict caffeine intake	Czigle & Tóth (2010b)
	Other systemic drugs for obstructive airway diseases, xanthines	Theophylline	CYP1A2		**↑** plasma level (23–29%) **↑** adverse reactions **!** reduce/restrict caffeine intake	Carrillo & Benitez (2000), Chrościńska-Krawczyk et al. (2014)
All other therapeutic products (V03)	Nerve depressants	Ethanol	CYP2D6		**↑** caffeine plasma level **!** reduce/restrict caffeine intake	Mohamed et al. (2011), Striley & Khan (2014)

**Note:**

ATC, Anatomical Therapeutic Chemical Classification System (World Health Organization, 2023); **↑,** increase (of); **↓,** decrease (of); !, warning; excl., excluding.

## Drug interactions of Ginkgonis folium

*Ginkgo biloba* L. (Ginkgoaceae), commonly also known as ginkgo or the maidenhair tree, is a species of dioecious tree native to China (Bensky, Gamble & Bensky, 1986; Czigle et al., 2018; van Beek, 2000).

The European Pharmacopoeia lists a monograph for the dried leaf of *Ginkgo biloba* L. (Ginkgonis folium) (European Pharmacopoeia, 2023c) and requires the content of not less than 0.5% flavonoids, expressed as flavone glycosides (M_r_ 757). The pharmacopoeial monograph for the refined and quantified dry extract produced from *Ginkgo* leaf (Ginkgonis extractum siccum raffinatum et quantificatum) (European Pharmacopoeia, 2023b) contains three positive criteria on active constituents: 22.0–27.0% flavone glycosides (M_r_ 756.7), 2.8–3.4% ginkgolides (A, B and C), and 2.6–3.2% bilobalide. The fourth criterion requires that the content of ginkgolic acids is less than 5 ppm (European Pharmacopoeia, 2023b).

Products prepared from *Ginkgo* leaf and from its refined and quantified dry extract, often marketed as EGb 761, are top-selling herbal medicinal products, especially in Europe, as well as important major botanical food supplements on the world market. The quality of these food supplements on the European market is often dubious and prone to adulteration (Czigle et al., 2018). In western allopathic medicine, *Ginkgo biloba* medications underwent strict evaluations of their clinical therapeutic use, for example, by the European Medicines Agency (EMA), with the following results: the *Ginkgo* leaf herbal medicinal dry extract is indicated in the well-established medicinal use category ‘for the improvement of (age-associated) cognitive impairment and of quality of life in mild dementia’, (European Medicines Agency, 2012d), ATC code: N06DX02 (World Health Organization, 2023), and the powdered *Ginkgo* leaf herbal substance is beneficial as a traditional herbal medicinal product ‘for the relief of heaviness of legs and the sensation of cold hands and feet associated with minor circulatory disorders’ (European Medicines Agency, 2012d). The European Union herbal monograph is accompanied by a detailed assessment report (European Medicines Agency, 2012b).

The indications for *G. biloba* leaf are well supported by in-depth studies on a number of defined chemical constituents. The best known are flavonoids (flavonols, *e.g*., quercetin, kaempferol, isorhamnetin and their mono-, di-, and triglycosides; biflavonoids, *e.g*., amentoflavone, ginkgetin, bilobetin, sciadopitysin) (Hasler et al., 1992; Hasler & Meier, 1993; Meier, Hasler & Sticher, 1992) and terpene lactones (several diterpenes, *e.g*., ginkgolides A, B, C, D, K, L, M, N, P, Q, and the pentanorditerpene bilobalide) (van Beek, 2005; van Beek & Lelyveld, 1992; van Beek & Montoro, 2009), but they also include hypersensitive (ginkgolic acid) (Fuzzati, Pace & Villa, 2003; van Beek & Wintermans, 2001) and neurotoxic compounds (ginkgotoxin) (Leistner & Drewke, 2010).

Ginkgolides are inhibitors of CYP1A2, CYP2C9, CYP2C19, CYP2D6, CYP2E1, CYP3A4 (Czigle & Tóth, 2009a, 2016; Williamson, Driver & Baxter, 2009, 2013) and of the P-gp (causing decreased absorption and increased elimination) (Czigle & Tóth, 2009a, 2016; Williamson, Driver & Baxter, 2009, 2013). Ginkgolides are also antagonists of the platelet-activating factor (PAF); (Czigle & Tóth, 2009a, 2016; Czigle et al., 2018; Oberpichler et al., 1990; Smith, Maclennan & Darlington, 1996), *Ginkgo* flavonoids affect hemorheology (Czigle & Tóth, 2016; Chen et al., 2019a; Witte, Anadere & Walitza, 1992) and vasopressin level (Czigle & Tóth, 2016; Kubota et al., 2006). The EGb 761 extract increases the synaptosomal uptake of 5-hydroxytryptamine (Czigle & Tóth, 2016; Ramassamy et al., 1992), acts as an GABA_A_ antagonist (Czigle & Tóth, 2016; Ivic et al., 2003), and has a neuroprotective effect (Ahlemeyer & Krieglstein, 2003). Biflavones (ginkgetin, isoginkgetin, bilobetin, and amentoflavone), and some flavonoids (apigenin, quercetin, isorhamnetin, luteolin, and kaempferol) inhibit thrombin activity (Chen et al., 2019c).

This review presents and summarizes relevant and more recent data on the drug interaction potential of *Ginkgo* leaf and its active metabolites. Interactions can occur at both the pharmacokinetic and pharmacodynamic levels (Table 2). *Ginkgo biloba* can increase the effects of anticoagulants, such as coumarin derivatives (European Medicines Agency, 2012d); patients with concomitant anticoagulant (*e.g*., warfarin, phenprocoumon) and antiplatelet treatment (*e.g*., clopidogrel, acetylsalicylic acid, and other non-steroidal anti-inflammatory drugs (NSAIDs)) should only use *Ginkgo* medicinal products after consultation with a physician (European Medicines Agency, 2012d). Concomitant use of *Ginkgo biloba* preparations and efavirenz (a non-nucleoside reverse transcriptase inhibitor) is not recommended, as plasma concentrations of efavirenz can be decreased due to induction of CYP3A4 (European Medicines Agency, 2012d). An interaction study with talinolol (a selective beta-blocking agent) indicates that *Ginkgo biloba* may inhibit P-gp (European Medicines Agency, 2012d). This can cause increased exposure of drugs markedly affected by the P-gp in the intestine, such as dabigatran etexilate (a direct thrombin inhibitor); caution is advised for the combination of *Ginkgo biloba* with dabigatran (European Medicines Agency, 2012d). A study of interaction has indicated that the C_max_ of nifedipine (a calcium channel blocker) increased by up to 100% in some individuals that resulted in dizziness and an increased severity of hot flashes (European Medicines Agency, 2012d).

**Table 2:** Drug interactions of Ginkgonis folium.

ATC therapeutic subgroup	ATC pharmacological/chemical subgroup	Drugs	Pharmacokinetic basis of interaction	Pharmacodynamic basis of interaction	Manifestation of interaction	
Stomatological preparation, in dentistry (A01)	Salicylic acid and derivatives	Acetylsalicylic acid	CYP1A2 CYP2C9 CYP3A4	EGb 761 is a PAF antagonist.	synergistic effect **↑** antiplatelet effect **!** bleeding risk (hyphema)	Ke et al. (2021)
Antithrombotic agents (B01)						
Analgetics (N02)						
Drugs for acid related disorders (A02)	H_2_-receptor antagonists	Cimetidine	CYP2C9	EGb 761 has a mucoprotective effect.	**↑** mucoprotective effect	Wang, Zhao & Ma (2000)
Drugs used in diabetes (A10)	Insulins and analogues	Insulin		EGb 761 increases the function of Langerhans islets β cells.	additive effect ! patient monitoring necessary	Banin et al. (2014)
Antithrombotic agents (B01)	Platelet aggregation inhibitors excl. heparin	Clopidogrel Dipyridamol Ticlopidine	CYP1A2 CYP2D6 P-gp	EGb 761 is a PAF antagonist.	synergistic effect **↑** antithrombotic effect **!** bleeding risk (hyphema)	Williamson, Driver & Baxter (2013, 2018)
	Direct thrombin inhibitors	Dabigatran etexilate		EGb 761 is a PAF antagonist.	additive effect ! bleeding risk	Williamson, Driver & Baxter (2013, 2018)
	Vitamin K antagonists	Warfarin	CYP1A2 CYP2C9	EGb 761 affects hemorheology.	additive effect ! bleeding risk	Stoddard et al. (2015)
	Heparin group	Dalteparin Enoxaparin Heparin	CYP1A2 CYP2D6 CYP3A4	EGb 761 affects hemorheology.	additive effect ! bleeding risk	Williamson, Driver & Baxter (2009, 2018).
Cardiac therapy (C01)	Cardiac glycosides	Digoxin	CYP3A4		**↑** plasma level ! hypokalemia	Mauro et al. (2003)
Diuretics (C03)	Low-ceiling diuretics, thiazides/sulfonamides	Hydrochlorothiazide Chlorothiazide Indapamide		EGb 761 affects vasopressin level.	**↓** diuretic effect ! hypertension risk	Williamson, Driver & Baxter (2013, 2018)
Calcium channel blockers (C08)	Selective calcium channel blockers with mainly vascular effects, or with direct cardiac effects	Nicardipine Nifedipine Verapamil	CYP3A4 CYP1A2		**↑** plasma level ! do not use combination	Williamson, Driver & Baxter (2013, 2018)
Immunosuppressants (L04)	Calcineurin inhibitors	Ciclosporin	CYP3A4 P-gp		**↑** plasma level	Williamson, Driver & Baxter (2013, 2018)
Anti-inflammatory and antirheumatic products (M01)	Anti-inflammatory and antirheumatic products, non-steroids	Celecoxib Diclofenac Ibuprofen Indometacin Naproxen Piroxicam	CYP2C9	EGb 761 is a PAF antagonist.	**!** gastric bleeding risk	Williamson, Driver & Baxter (2009, 2018)
Antiepileptics (N03)	Antiepileptics, carboxamide derivatives	Carbamazepine	CYP3A4 CYP2C8	Neurotoxic ginkgotoxin (mainly in seeds).	**↓** anticonvulsive effect	Williamson, Driver & Baxter (2013, 2018)
	Antiepileptics, fatty acid derivatives	Valproic acid	CYP2C9 CYP2A6	Neurotoxic ginkgotoxin (mainly in seeds).	**↓** anticonvulsive effect	Williamson, Driver & Baxter (2009, 2013, 2018)
	Antiepileptics, barbiturates and derivatives	Phenobarbital	CYP2C9	Neurotoxic ginkgotoxin (mainly in seeds).	**↓** anticonvulsive effect	Czigle & Tóth (2016), Williamson, Driver & Baxter (2013, 2018)
	Antiepileptics, hydantoin derivatives	Primidone Phenytoin Gabapentin		Neurotoxic ginkgotoxin (mainly in seeds).	**↓** anticonvulsive effect	Williamson, Driver & Baxter (2009, 2018)
Psycholeptics (N05)	Antipsychotics	Haloperidol	CYP2D6		**↑** antipsychotic effect	Zhang et al. (2001)
	Anxiolytics, benzodiazepine derivatives	Alprazolam Diazepam	Absorption CYP2C9 CYP3A4		**↑** anxiolytic effect	Williamson, Driver & Baxter (2009, 2018), Zuo et al. (2010)
	Anxiolytic drugs, azaspirodecanedione derivatives	Buspirone	absorption		**↓** absorption **↓** anxiolytic effect	Spinella & Eaton (2002)
	Hypnotics and sedatives, benzodiazepine derivatives	Midazolam				Williamson, Driver & Baxter (2009, 2018)
Psychoanaleptics (N06)	Antidepressants; monoamine oxidase inhibitors, non-selective; selective serotonin reuptake inhibitors; other antidepressants	Tranylcypromin Trazodon Fluoxetine	CYP3A4 CYP2C19 CYP2D6	EGb 761 is a serotonin inhibitor; and GABA_A_-agonist.	**↑** antidepressant/sedative effect **↑** adverse reaction (coma risk)	Czigle & Tóth (2016)
	Psychostimulants, agents used for ADHD and nootropics	Caffeine	CYP1A2		**↑** psychostimulant effect	Czigle & Tóth (2016)

**Note:**

ATC, Anatomical Therapeutic Chemical Classification System (WHO, 2023); **↑,** increase (of); **↓,** decrease (of); !, warning; EGb 761, refined and quantified dry extract produced from *Ginkgo* leaf; GABA, γ-aminobutyric acid; PAF, platelet-activating factor; P-gp, P-glycoprotein.

## Drug interactions of Valerianae radix

Valerian is an old favorite in European phytotherapy. The monograph of valerian root (Valerianae radix) in the European Pharmacopoeia describes the dried, whole or fragmented underground parts of *Valeriana officinalis* L. *s. l*. (Valerianaceae), including the rhizome surrounded by the roots and stolons (European Pharmacopoeia, 2023o). The pharmacopoeia also lists further monographs with pharmaceutical quality used in human medicine: valerian root, cut for the purpose of being used in herbal teas (Valerianae radix minutata) (European Pharmacopoeia, 2023p), valerian dry hydroalcoholic extract (Valerianae extractum hydroalcoholicum siccum) (European Pharmacopoeia, 2023n), valerian dry aqueous extract (Valerianae extractum aquosum siccum) (European Pharmacopoeia, 2023m), and valerian tincture (Valerianae tinctura) (European Pharmacopoeia, 2023q).

Recently, *Valeriana officinalis* medications underwent evaluations of their clinical therapeutic use, *e.g*., by the European Medicines Agency, with the following results: the dry extract of Valerianae radix is indicated in the category of well-established medicinal use for the ‘relief of mild nervous tension and sleep disorders’, (European Medicines Agency, 2015c), ATC code: N05CM09 (World Health Organization, 2023), and as a traditional medicinal product Valerianae radix and its dry extracts, tinctures, and expressed juice (from fresh root), are beneficial for the ‘relief of mild symptoms of mental stress and to aid sleep’, (European Medicines Agency, 2015c). The valerian essential oil (Valerianae aetheroleum), as a traditional herbal medicinal product is beneficial for the ‘relief of mild symptoms of mental stress and to aid sleep’, (European Medicines Agency, 2015b). This review article only presents relevant and more recent data that are not part of these herbal monographs or related assessment reports (European Medicines Agency, 2015a).

The combination of valerian root and hop strobile (Valerianae radix and Lupuli flos) is indicated in the category of well-established medicinal use for the ‘relief of sleep disorders’, and in the category of traditional use for the ‘relief of mild symptoms of mental stress’, and ‘to aid sleep’, (European Medicines Agency, 2017b).

Sedative herbal tea combinations (Species sedativae) shall contain 2, 3 or 4 herbal substances out of hop strobile—Lupuli flos (*Humulus lupulus* L., Cannabaceae), lavender flower—Lavandulae flos (*Lavandula angustifolia* Mill., Lamiaceae), *Melissa* leaf—Melissae folium (*Melissa officinalis* L., Lamiaceae), passionflower herb—Passiflorae herba (*Passiflora incarnata* L., Passifloraceae), and valerian root—Valerianae radix (*Valeriana officinalis* L., Valerianaceae). Depending on the chosen herbal substances, the percentage of Valerianae radix in combination is 15–57% or 33–57%. This herbal tea is beneficial as a traditional herbal medicinal product for the ‘relief of mild symptoms of mental stress and to aid sleep’, (European Medicines Agency, 2017a).

The major biologically active compounds of Valerianae radix are flavonoids, iridoids, lignans, volatile compounds, such as monoterpenes (borneol, bornyl acetate), and sesquiterpenes (valerenone, valerenic and acetoxyvalerenic acids) (Czigle & Tóth, 2010a, 2016; Orhan, 2021).

A possible mechanism, by which Valerianae radix may cause sedation, is by increasing the synaptic amount of γ-aminobutyric acid (GABA) (Johnston et al., 2006; Orhan, 2021; Savage et al., 2018). Flavonoids (Wasowski & Marder, 2012) and monoterpenes (bornyl acetate) (Wang & Heinbockel, 2018) have GABA agonistic properties, and flavonoids also act as MAO inhibitors (Dhiman et al., 2019). Valerenic acid could attenuate increases in serum corticosterone levels in a mouse model of stress, the extract could modulate serotonin (5-HT) and norepinephrine (NE) turnover in the hippocampus and amygdala region (Jung et al., 2015). Valerenic acid is a selective positive allosteric modulator of the GABA_A_ receptor (Khom et al., 2007), an agonist at the metabotropic glutamate receptor mGlu I, but an antagonist at the mGlu II (Del Valle-Mojica & Ortíz, 2012; Feinberg et al., 2005); a partial agonist at the 5-HT_5A_ receptor (Dietz et al., 2005), it also has antidepressant potential. Isovaltrate (a valepotriate) is an inverse agonist at the adenosine A_1_ receptor (Sichardt et al., 2007), and it presents antidepressant-like activity, mediated by dopaminergic, noradrenergic, serotonergic neurotransmissions (Dietz et al., 2005; Jayaraj et al., 2020; Orhan, 2021; Ortiz, Nieves-Natal & Chavez, 1999). It also has inhibitory activity towards the Na^+^/K^+^-ATPase (Müller et al., 2015). Some lignans, for example 1-hydroxypinoresinol, are GABA_A_ receptor agonists; and 4′-*O*-β-d-glucosyl-9-*O*-(6″-deoxysaccharosyl)olivil is a partial agonist at adenosine A_1_ receptors (Cavadas et al., 1995; Savage et al., 2018; Schumacher et al., 2002).

Interactions occur at the pharmacokinetic and pharmacodynamic levels (Table 3). Different preparations of valerian root have been proven to inhibit the cytochrome P_450_ isoenzyme CYP3A4. In contrast, *Valeriana* does not affect other cytochrome P_450_ isoenzymes CYP1A2, CYP2C9, CYP2C19, CYP2D6 and CYP2E1 (Bogacz et al., 2014; Czigle & Tóth, 2010a, 2016; Williamson, Driver & Baxter, 2009, 2013, 2018). Pharmacodynamic interactions are related to the mechanism of action of secondary metabolites. *Valeriana* does not affect the pharmacokinetics of caffeine, alprazolam, or midazolam to a clinically relevant extent (European Medicines Agency, 2015c). The stimulant effect of caffeine may oppose the hypnotic effects of valerian (European Medicines Agency, 2015c). However, additive anxiolytic effects are possible with benzodiazepines (European Medicines Agency, 2015c).

**Table 3:** Drug interactions of Valerianae radix.

ATC therapeutic subgroup	ATC pharmacological/chemical subgroup	Drugs	Pharmacokinetic basis of interaction	Pharmacodynamic basis of interaction	Manifestation of interaction	
Antidiarrheals, intestinal anti-inflammatory/antiinfective agents (A07)	Antipropulsives	Loperamide	CYP3A4	*Valeriana* is a GABA_A_ agonist, mGlu I agonist, mGlu II antagonist, MAO inhibitor, 5-HT_5A_ agonist, A_1_ agonist.	additive effect **↑** adverse reaction	Czigle & Tóth (2016), Kelber, Nieber & Kraft, 2014)
Lipid modifying agents (C10)	HMG CoA reductase inhibitors	Lovastatin	CYP3A4 P-gp		**↑** plasma level **↑** adverse reaction (rabdomyolysis)	Czigle & Tóth (2016)
Antifungals for dermatological use (D01)	Antifungals for topical use, imidazole and triazole derivatives	Ketoconazole	CYP3A4		**↑** plasma level **↑** adverse reactions	Czigle & Tóth (2016), Hellum & Nilsen (2008)
Antimycotics for systemic use (J02)	Triazole and tetrazole derivatives	Itraconazole	CYP3A4		**↑** plasma level **↑** adverse reactions	Czigle & Tóth (2016), Hellum & Nilsen (2008)
Antineoplastic agents (L01)	Plant alkaloids and other natural products	Etoposide Paclitaxel Vinblastine Vincristine Vindesine	CYP3A4		**↑** plasma level **↑** adverse reactions	Czigle & Tóth (2016), Mooiman et al. (2014)
Anesthetics (N01)	Anesthetics, general	Propofol Thiopental		*Valeriana* is a GABA_A_ agonist, mGlu I agonist, mGlu II antagonist, MAO inhibitor, 5-HT_5A_ agonist, A_1_ agonist.	additive effect **↑** adverse reactions **!** do not use combination	Czigle & Tóth (2016)
Analgesics (N02)	Opioids	Fentanyl Morphine		*Valeriana* is a GABA_A_ agonist, mGlu I agonist, mGlu II antagonist, MAO inhibitor, 5-HT_5A_ agonist, A_1_ agonist.	additive effect **↑** adverse reactions **!** do not use combination	Czigle & Tóth (2016)
Antiepileptics (N03)	Antiepileptics, barbiturates	Phenobarbital	CYP2C9	*Valeriana* is a GABA_A_ agonist, mGlu I agonist, mGlu II antagonist, MAO inhibitor, 5-HT_5A_ agonist, A_1_ agonist.	additive effect **↑** adverse reactions **!** do not use combination	Czigle & Tóth (2016), Dietz et al. (2005)
	Antiepileptics, benzodiazepine derivatives	Clonazepam	CYP1A2 CYP3A4	*Valeriana* is a GABA_A_ agonist, mGlu I agonist, mGlu II antagonist, MAO inhibitor, 5-HT_5A_ agonist, A_1_ agonist.	additive effect **↑** adverse reaction **!** do not use combination	Dietz et al. (2005), Kelber, Nieber & Kraft (2014)
Psycholeptics drugs (N05)	Anxiolytic drugs, benzodiazepine derivatives, azaspirodecanedione derivatives	Alprazolam Diazepam Lorazepam Buspirone	CYP2C9 CYP3A4	*Valeriana* is a GABA_A_ agonist, mGlu I agonist, mGlu II antagonist, MAO inhibitor, 5-HT_5A_ agonist, A_1_ agonist.	**↑** alprazolam plasma level (19%) **↑** anxiolytic effect **!** do not use combination	Cavadas et al. (1995), Dietz et al. (2005), Kelber, Nieber & Kraft (2014)
	Hypnotics and sedatives, barbiturates	Pentobarbital Secobarbital	CYP2C9	*Valeriana* is a GABA_A_ agonist, mGlu I agonist, mGlu II antagonist, MAO inhibitor, 5-HT_5A_ agonist, A_1_ agonist.	additive effect **↑** adverse reaction (hypotension) **!** do not use combination	Cavadas et al. (1995), Czigle & Tóth (2016), Dietz et al. (2005)
	Hypnotics, benzodiazepine derivatives	Midazolam Temazepam Triazolam	CYP2C19 CYP3A4	*Valeriana* is a GABA_A_ agonist, mGlu I agonist, mGlu II antagonist, MAO inhibitor, 5-HT_5A_ agonist, A_1_ agonist.	**↑** plasma level **↑** anxiolytic effect **!** do not use combination	Cavadas et al. (1995), Czigle & Tóth (2016), Dietz et al. (2005), Mooiman et al. (2014)
Antihistamines for systemic use (R06)	Selective peripheral H_1_ blocker	Fexofenadine	CYP3A4 Pgp OATP	*Valeriana* is a GABA_A_ agonist, mGlu I agonist, mGlu II antagonist, MAO inhibitor, 5-HT_5A_ agonist, A_1_ agonist.	**↑** plasma level **↑** adverse reactions (sedative/hypnotic effect)	Czigle & Tóth (2016), Dietz et al. (2005)

**Note:**

ATC, Anatomical Therapeutic Chemical Classification System (WHO, 2023); **↑,** increase (of); !, warning; A_1_ receptor, adenosine receptor, subtype A_1_; GABA, γ-aminobutyric acid; mGlu I, mGlu II receptors, metabotropic glutamate receptors subtype I a II; 5-HT_5A_ receptor, 5-hydroxytryptamine (serotonin) 5A subtype receptor; MAO, monoamine oxidase; H, histamine; OATP, organic anion transporters; P-gp, P-glycoprotein.

## Drug interactions of Hyperici herba

The herbal drug Hyperici herba consists of whole or fragmented, dried flowering tops of St. John’s wort (*Hypericum perforatum* L., Hypericaceae), harvested during flowering time (European Pharmacopoeia, 2023l). The Pharmacopoeia also lists a monograph for the St. John’s wort dry extract, quantified (Hyperici herbae extractum siccum quantificatum) (European Pharmacopoeia, 2023k) with pharmaceutical quality used in human medicine. In Western allopathic medicine, *Hypericum perforatum* medications were subjected to strict evaluations of their clinical therapeutic use, *e.g*., by the European Medicines Agency, with the following results: the *Hypericum* dry extract is indicated in the category of well-established medicinal use for the ‘treatment of mild to moderate depressive episodes, and for the short term treatment of symptoms in mild depressive disorders’, (European Medicines Agency, 2021), ATC code: N06AX25 (World Health Organization, 2023), and as a traditional medicinal product the Hyperici herba dry and liquid extracts, tinctures, expressed juice (from the fresh herb), and the comminuted herbal substance are beneficial for the ‘relief of temporary mental exhaustion, for the symptomatic treatment of minor inflammations of the skin (such as sunburn) and as an aid in healing of minor wounds, as well as for the symptomatic relief of mild gastrointestinal discomfort’, (European Medicines Agency, 2021). This review article presents relevant and more recent data that are not part of the European Union herbal monograph or the related assessment report (European Medicines Agency, 2016a).

The final antidepressant effect arises on the basis of the synergy of different biologically active compounds (Czigle & Tóth, 2009b; Nagy, Mučaji & Grančai, 2017). The phloroglucinol derivatives hyperforin and adhyperforin are re-uptake inhibitors of serotonin, dopamine, and norepinephrine (Tian et al., 2014). Hyperforin is a transient receptor potential channels (TRPC6) activator as well, and it induces the release of calcium and zinc from mitochondria (Tu, Gibon & Bouron, 2010). Recent data showed that the increase in the intracellular concentration of calcium ions is mediated by the activation of the mentioned TRPC6, a diacylglycerol-sensitive C-class of transient receptor potential channels, and without the activation of other isoforms (TRPC1, TRPC3, TRPC4, TRPC5, and TRPC7) (Nagy, Mučaji & Grančai, 2017; Tu, Gibon & Bouron, 2010). Hyperforin is also a GABA reuptake inhibitor (Zanoli, 2004). Flavonoids and proanthocyanins have a GABA agonistic effect (Hanrahan, Chebib & Johnston, 2011). The naphthodianthrone derivative hypericin is a MAO-A and MAO-B inhibitor (Butterweck, 2003), and dopamine β-hydroxylase inhibitor (Kleber et al., 1999), leading to increased dopamine levels, although thus possibly decreasing norepinephrine and epinephrine. It also affects 5-HT_1A_ (Teufel-Mayer & Gleitz, 1997) and D_3_ receptors (McClatchey et al., 2009), and is a β-sympathomimetic (Nagy, Mučaji & Grančai, 2017).

Interactions occur at both the pharmacokinetic and pharmacodynamic levels. The *Hypericum* dry extract induces the expression of CYP3A4, CYP2C9, CYP2C19 (Czigle & Tóth, 2009b, 2016; Williamson, Driver & Baxter, 2009, 2013, 2018), which results in increased drug metabolism; it also inhibits the P-glycoprotein (causing decreased oral absorption and increased elimination) (Czigle & Tóth, 2009b, 2016; Dürr et al., 2000; Williamson, Driver & Baxter, 2013). Special care should be taken in the case of concomitant use of all drug substances whose kinetics are influenced by CYP3A4, CYP2C9, CYP2C19, or P-gp (*e.g*., digoxin, simvastatin, methadone, benzodiazepine derivatives, amitriptyline, finasteride, fexofenadine), because a reduction in plasma concentrations of these drugs is possible (Table 4). Concomitant use of immunosuppressive drugs for systemic use (cyclosporine/ciclosporin, tacrolimus) (Alscher & Klotz, 2003; Czigle & Tóth, 2016), antivirals for systemic use (protease inhibitors, such as amprenavir, indinavir, and others), several antineoplastic agents, such as topoisomerase 1 inhibitors (irinotecan), and anticoagulants, vitamin K antagonists (warfarin) is contraindicated (Table 4). A reduction in plasma concentrations of hormonal contraceptives for systemic use can lead to increased intermenstrual (breakthrough) bleeding and to a failure of the contraceptive effect. Women who use oral contraceptives should take alternate or additional contraceptive measures (Table 4) (Czigle & Tóth, 2016; European Medicines Agency, 2021; Hall et al., 2003).

**Table 4:** Drug interactions of Hyperici herba.

ATC therapeutic subgroup	ATC pharmacological/chemical subgroup	Drugs	Pharmacokinetic basis of interaction	Pharmacodynamic basis of interaction	Manifestation of interaction	
Drugs for acid related disorders (A02)	Proton pump inhibitors	Omeprazole	CYP2C19 CYP3A4		**↓** plasma level	Borrelli & Izzo (2009)
Antithrombotic agents (B01)	Platelet aggregation inhibitors excl. heparin	Clopidogrel	CYP1A2 CYP2D6		**↑** active metabolites plasma level **↑** antithrombotic effect **!** adverse reaction (bleeding)	Lau et al. (2011)
	Vitamin K antagonists	Warfarin Phenprocoumon	CYP1A2 CYP2C9 CYP3A4 CYP2C19		**↓** INR **↑** PT **↓** anticoagulant effect	Borrelli & Izzo (2009)
Cardiac therapy (C01)	Cardiac glycosides	Digoxin	CYP3A4 Pgp		**↓** plasma level	Borrelli & Izzo (2009)
Antihypertensives (C02)	Antiadrenergic agents, centrally acting (*Rauvolfia* alkaloids)	Reserpine	CYP1A2 CYP2C6 CYP2D1 CYP2D2 CYP2E1 CYP3A4	*Hypericum* is a serotonin reuptake and MAO inhibitor.	antagonistic effect	Nicolussi et al. (2020)
Calcium channel blockers (C08)	Selective calcium channel blockers with direct cardiac effects	Verapamil	CYP3A4 CYP1A2		**↓** plasma level	Tannergren et al. (2004)
Lipid modifying agents (C10)	HMG CoA reductase inhibitors	Simvastatin Pravastatin Fluvastatin Atorvastatin Lovastatin	CYP3A4 P-gp		**↓** plasma level	Eggertsen, Andreasson & Andrén (2007)
Sex hormones and modulators of the genital system (G03)	Hormonal contraceptives for systemic use	Ethinylestradiol (EE), Estradiol + Progestogens	CYP1A2 CYP2C9 CYP3A4		**↓** estrogen (EE) plasma level (13–15%) **!** breakthrough bleeding **!** contraceptive failure **!** alternate methods of contraception are advised.	Borrelli & Izzo (2009), Hall et al. (2003)
Urologicals (G04)	Testosterone-5α reductase inhibitors	Finasteride	CYP3A4 P-gp		**↓** plasma level	Lundahl et al. (2009)
Antivirals for systemic use (J05)	Protease inhibitors	Amprenavir Indinavir Nelfinavir Ritonavir Saquinavir	CYP3A4 P-gp		**↓** plasma level **↓** AUC (57%) **↑** elimination	Borrelli & Izzo (2009), James (2000)
	Non-nucleoside reverse-transcriptase inhibitors	Nevirapine Delavirdine Efavirenz	CYP3A4 P-gp		**↓** plasma level	Borrelli & Izzo (2009)
Antineoplastic agents (L01)	BCR-ABL tyrosine kinase inhibitors	Imatinib	CYP3A4		**↓** plasma level	Smith et al. (2004)
	Topoisomerase 1 inhibitors	Irinotecan	CYP3A4		**↓** plasma level **↓** AUC (42%)	Mathijssen et al. (2002)
	Sensitizers used in photodynamic/radiation therapy	Aminolevulinic acid	up-regulation of CYP activity	Hypericin is phototoxic.	**↑** light-induced toxicity (15%) **!** Synergistic photensitivity reaction between aminolevulinic acid and hypericin.	Boiy, Roelandts & de Witte (2011), Ladner et al. (2001), Ritz et al. (2012), Schneider-Yin et al. (2009)
Endocrine therapy (L02)	Anti-estrogens	Tamoxifen	CYP2C9		**↓** plasma level	Hansten (2018)
Immunosuppressants drugs (L04)	Calcineurin inhibitors	Cyclosporine Tacrolimus	CYP3A4 P-gp		**↓** cyclosporine (30–70%), and tacrolimus plasma level **!** transplant rejection risk	Alscher & Klotz (2003), Borrelli & Izzo (2009), Hebert et al. (2004)
Analgesics (N02)	Opioids, benzomorphan derivatives	Pentazocine	CYP3A4	*Hypericum* is a serotonin reuptake and MAO inhibitor, GABA agonist	additive serotonergic effect **!** risk: serotonin syndrome or Call-Fleming syndrome	Drugs (2023)
	Other opioids	Tramadol	CYP1A2 CYP3A4	*Hypericum* is a serotonin reuptake and MAO inhibitor, GABA agonist.	additive serotonergic effect **!** risk: serotonin syndrome or Call-Fleming syndrome	Sansone & Sansone (2009)
	Drugs used in opioid dependence Opioids, diphenylpropylamine derivatives	Methadone	CYP3A4 CYP2C19 CYP2D6	*Hypericum* is a serotonin reuptake and MAO inhibitor, GABA agonist.	**↑** methadone effect **↑** adverse reactions **!** coma risk	Borrelli & Izzo (2009)
	Other analgesics and antipyretics, anilides	Paracetamol	CYP1A2 CYP3A4 CYP2E1		**↑** analgetic effect	Jiang et al. (2022)
	Antimigraine preparations, selective serotonin (5-HT1) agonists	Frovatriptan Naratriptan Rizatriptan Sumatriptan Zomitriptan	CYP1A2 CYP3A4 CYP2D6 MAO-A P-gp	*Hypericum* is a serotonin reuptake and MAO inhibitor, GABA agonist.	additive serotonergic effect **!** risk: serotonin syndrome or Call-Fleming syndrome	Bonetto et al. (2007)
Antiepileptics (N03)	Antiepileptics, barbiturates and derivatives	Phenobarbital Pentobarbital Secobarbital	CYP2C9		**↓** anticonvulsive effect **!** dosage adjustment	Nicolussi et al. (2020)
	Antiepileptics, hydantoin derivatives	Phenytoin	CYP2C9 CYP2C19		**↓** anticonvulsive effect **!** dosage adjustment	Borrelli & Izzo (2009)
	Antiepileptics, carboxamide derivatives	Carbamazepine	CYP3A4		**↓** anticonvulsive effect **!** dosage adjustment	Borrelli & Izzo (2009)
Psycholeptics (N05)	Anxiolytics, benzodiazepine derivatives	Alprazolam Diazepam	CYP2C19 CYP3A4		**↓** anxiolytic effect	Borrelli & Izzo (2009)
	Hypnotics and sedatives, benzodiazepine derivatives	Midazolam	CYP2C19 CYP3A4		**↓** hypnotic effect	Borrelli & Izzo (2009)
Psychoanaleptics (N06)	Antidepressants, non-selective monoamine reuptake inhibitors	Amitriptylin Nortriptylin	CYP3A4 CYP2C9 CYP2D6 P-gp	*Hypericum* is a serotonin reuptake and MAO inhibitor, GABA agonist.	**↓** plasma level of amitriptylin (22%) and its metabolite nortriptylin (42%) **↓** antidepressant effect	Borrelli & Izzo (2009)
	Antidepressants, MAO inhibitors, non-selective	Tranylcypromine	MAO		additive effect **↑** adverse reaction **!** 14 days interval	Sacher et al. (2011)
	Antidepressants, selective serotonin reuptake inhibitors	Paroxetine Sertraline	CYP2D6 CYP3A4		additive serotonergic effect **!** risk: serotonin syndrome or Call-Fleming syndrome	Cui & Zheng (2016)
	Antidepressants, other antidepressants	Nefazodone	CYP2D6 CYP3A4		additive serotonergic effect **!** risk: serotonin syndrome or Call-Fleming syndrome	Borrelli & Izzo (2009)
Drugs for obstructive airway diseases (R03)	Other systemic drugs for obstructive airway diseases, xanthines	Aminophylline Theophylline	CYP1A2		**↓** plasma level	Morimoto et al. (2004)
Cough and cold preparations (R05)	Cough suppressants, excl. combinations with expectorants	Dextromethorphan	CYP3A4 CYP2D6	*Hypericum* is a serotonin reuptake and MAO inhibitor, GABA agonist.	additive serotonergic effect **!** risk: serotonin syndrome or Call-Fleming syndrome	Markowitz et al. (2000)
Antihistamines for systemic use (R06)	Other antihistamines for systemic use	Fexofenadine	CYP3A4 P-gp OATP		**↑** plasma level **↑** adverse reactions	Wang et al. (2002)

**Note:**

ATC, Anatomical Therapeutic Chemical Classification System (WHO, 2023); **↑,** increase (of); **↓,** decrease (of); !, warning; excl., excluding; AUC, area under the curve; GABA, γ-aminobutyric acid; INR, international normalized ratio; MAO, monoamine oxidase; OATP, organic anion transporters; P-gp, P-glycoprotein; PT, prothrombin time.

The outcome of the pharmacodynamic interaction is the risk of serotonin syndrome (Czigle & Tóth, 2016; Spadaro et al., 2022; Volpi-Abadie, Kaye & Kaye, 2013) or Call-Fleming syndrome (Czigle & Tóth, 2016; Skandhan, Ramakrishnan & Anand, 2013). The reason is that the *Hypericum* dry extract increases serotonergic effects when combined with antidepressants affecting serotonin, such as selective serotonin reuptake inhibitors (*e.g*., sertraline, paroxetine, nefazodone), tricyclic antidepressants, selective serotonin (5-HT_1A_) agonists employed in anxiety treatment (buspirone), or selective serotonin (5-HT_1BD_) agonists used for acute migraine attacks (triptans) (Table 4). The serotonin syndrome (Volpi-Abadie, Kaye & Kaye, 2013) is a potentially life-threatening condition based on the overactivation of both the central postsynaptic and peripheral 5HT_1A_, and most notably the 5HT_2A_ receptors. Symptoms include a combination of altered cognitive functions (cephalgia, agitation, hypomania, confusion, hallucination, coma), autonomic hyperactivity (tremor, diaphoresis, hypertension, tachycardia, nausea, vomitus), neuromuscular hyperactivity (rigidity, tremor, myoclonus, hyperreflexia) and hyperthermia (Spadaro et al., 2022; Volpi-Abadie, Kaye & Kaye, 2013). Symptoms usually begin within 24 h of an increased dose of a serotonergic drug/agent, the addition of another serotonergic agent to a drug regimen, or overdosing. Most patients will seek help in a hospital in 6 h; however, patients with mild symptoms may have a more subacute or chronic presentation (Volpi-Abadie, Kaye & Kaye, 2013). The Call-Fleming syndrome (Skandhan, Ramakrishnan & Anand, 2013), also called reversible cerebral vasoconstriction syndrome (RCVS), is characterized by thunderclap headache and reversible vasoconstriction of the cerebral arteries.

During the treatment with *Hypericum* extracts, intense UV exposure should be avoided. Hypericin (a red pigment) ( $\lambda$ 595 nm) (Skalkos et al., 2006) is phototoxic (Schempp et al., 2000, 2001, 2003). A synergistic photosensitivity reaction has been observed between aminolevulinic acid (photodynamic therapy) (Boiy, Roelandts & de Witte, 2011; Ritz et al., 2012; Schneider-Yin et al., 2009) and hypericin (Table 4).

## Drug interactions of Lupuli flos

The pharmacopoeial herbal drug hop strobile (Lupuli flos), having pharmaceutical quality and used in human medicine, is described as the dried, generally whole, female inflorescence (strobile) of hop (*Humulus lupulus* L., Cannabaceae) (European Pharmacopoeia, 2023i). Hops (hop strobiles) are also commonly used culinarily as a bittering and flavoring agent in the production of beer. In allopathic medicine, *Humulus lupulus* medications underwent strict evaluations of their clinical therapeutic use, *e.g*., by the EMA with the following results: Lupuli flos dry and liquid extracts (ethanolic or sweet wine extract), tincture, as well as the comminuted or powdered herbal substance are indicated in the category of traditional herbal medicinal products that are beneficial for the ‘relief of mild symptoms of mental stress and to aid sleep’, (European Medicines Agency, 2013). This review article presents relevant and more recent data that are not included in the European Union herbal monograph on hop strobile and the related assessment report (European Medicines Agency, 2005).

The medicinal use of valerian root and hop strobile as a combination (Valerianae radix et Lupuli flos) is indicated in the category of well-established medicinal use for the ‘relief of sleep disorders’, and in the category of traditional use for the ‘relief of mild symptoms of mental stress and to aid sleep’, (European Medicines Agency, 2017b).

Sedative herbal tea combinations (Species sedativae) shall contain 2, 3 or 4 herbal substances out of hop strobile—Lupuli flos (*Humulus lupulus* L., Cannabaceae), lavender flower—Lavandulae flos (*Lavandula angustifolia* Mill., Lamiaceae), *Melissa* leaf—Melissa folium (*Melissa officinalis* L., Lamiaceae), passionflower herb—Passiflorae herba (*Passiflora incarnata* L., Passifloraceae), and valerian root—Valerianae radix (*Valeriana officinalis* L., Valerianaceae). Depending on the chosen herbal substances, the percentage of Lupuli flos in combination is 15–40%, 15–43%, or 21–36%. This herbal tea is beneficial as a traditional herbal medicinal product for the ‘relief of mild symptoms of mental stress and to aid sleep’, (European Medicines Agency, 2017a).

The final anxiolytic/hypnotic effect of hop strobile is based on the synergy of different biologically active compounds. Lupuli flos contains resins with phloroglucinols (α-acids (humulone and its derivates), β-acids (lupulone and its derivates), and their degradation product 2-methyl-3-buten-2-ol), flavonoids (xanthohumol), and terpenes (β-myrcene, β-caryophyllene) (Nagy, Mučaji & Grančai, 2017).

Some Lupuli flos components, including those contained in beer (non-alcoholic), are believed to act in the CNS by influencing GABA, adenosine, serotonin, and melatonin neurotransmission with an effective sedative action that both modulates the circadian rhythms and the sleep-wake cycle and is beneficial for the induction of sleep (Abourashed, Koetter & Brattström, 2004; Brattström, 2007; Butterweck et al., 2007; Dietz et al., 2005; Franco et al., 2012; Savage et al., 2018). Humulone is a positive allosteric modulator of GABA_A_ receptors (Benkherouf et al., 2020). Some components of hop resin (including the degradation product 2-methyl-3-buten-2-ol) increase GABA activity by modulating GABA_A_ receptors (Franco et al., 2012). The Lupuli flos extract is an agonist of the melatonin receptor MT_1_ but α- and β-acids do not affect melatonin receptors (Grundmann et al., 2006). Melatonin secretion has a circadian character, its receptors (MT_1_ and MT_2_) are localized in the suprachiasmatic nucleus; melatonin regulates circadian rhythms and hence the sleep-wake cycle in humans. *Humulus* active compounds have an α_2_-agonistic effect (β-myrcene) (Surendran et al., 2021), they affect the cannabinoid CB_2_ receptor (β-caryophyllene) (Alberti et al., 2017; Klauke et al., 2014) and opioid receptors (Park et al., 2012). Lupuli flos also contains several compounds with estrogenic activity, such as the prenylated flavonoid 6-prenylnaringenin (Carbone & Gervasi, 2022; Dietz et al., 2017; Hemachandra et al., 2012; Tan et al., 2014; Yoshimaru et al., 2014). Prenylflavonoids (xanthohumol, isoxanthohumol and 8-prenylnaringenin) are capable of modulating aromatase activity, decreasing estrogen synthesis (Monteiro et al., 2006).

Interactions occur at both the pharmacokinetic and pharmacodynamic levels. Lupuli extract is a CYP1A2, CYP2C8, CYP2C9, CYP2C19 inhibitor (Czigle & Tóth, 2016; Foster et al., 2011; TRC, 2023; Williamson, Driver & Baxter, 2013; Yuan et al., 2014). 8-Prenylnaringenin is an inhibitor of multidrug resistance-associated transporters, P-glycoprotein and MRP1 (Wesołowska et al., 2010). Abundant Phase II conjugates of the prenylated flavonoids were observed, including monoglucuronides, diglucuronides, monosulfates and sulfate-glucuronic acid deconjugates (van Breemen et al., 2020). No clinically significant interactions are reported in the European Union herbal monograph (European Medicines Agency, 2013). The interactions between *Humulus* and other drugs are based mainly on experimental evidence (European Medicines Agency, 2013; Williamson, Driver & Baxter, 2009, 2013, 2018) (Table 5).

**Table 5:** Drug interactions of Lupuli flos.

ATC therapeutic subgroup	ATC pharmacological/chemical subgroup	Drugs	Pharmacokinetic basis of interaction	Pharmacodynamic basis of interaction	Manifestation of interaction	
Endocrine therapy (L02)	Anti-estrogens	Tamoxifen	CYP2C9	*Humulus* has estrogenic effects (prenylated flavonoids).	antagonistic effect	Harrigan et al. (2021), Yoshimaru et al. (2014)
Analgesics (N02)	Other analgesics and antipyretics, anilides	Paracetamol	CYP1A2 CYP3A4 CYP2E1	β-Caryophyllene affects CB_2_ receptor	**↑** analgetic effect	Horvat et al. (2007), Jakovljevic et al. (2009)
Antiepileptics (N03)	Antiepileptics, barbiturates and derivatives	Phenobarbital	CYP3A4 CYP2C9	*Humulus* has an α_2_-agonistic effect (β-myrcene), affects CB_2_ (β-caryophyllene) and opioid receptors. Humulone is a positive allosteric modulator of GABA_A_ receptors.	additive effect **↑** adverse reactions	Raskovic et al. (2007), Raskovic et al. (2016)
Psycholeptics (N05)	Anxiolytics, benzodiazepine derivatives	Diazepam	CYP1A2 CYP2C19 CYP3A4	*Humulus* has an α_2_-agonistic effect (β-myrcene), affects CB_2_ (β-caryophyllene) and opioid receptors. Humulone is a positive allosteric modulator of GABA_A_ receptors.	additive effect **↑** adverse reactions	Raskovic et al. (2007, 2016)

**Note:**

ATC, Anatomical Therapeutic Chemical Classification System (WHO, 2023); **↑,** increase (of); **↓,** decrease (of); !, warning; CB_2_, cannabinoid receptor type 2; GABA_A_, γ-aminobutyric acid A receptor.

## Drug interactions of cannabinoids-containing herbal drugs (Cannabis flos, Cannabis herba, Cannabis resina)

Observing the impact of marijuana (Cannabis herba) or hashish (Cannabis resina) on the human body opened the way for the medical use of some cannabinoid receptor substrates in the therapy of certain diseases (*e.g*., as analgesics, antiemetics, antiepileptics) (Czigle & Tóth, 2016; Hill et al., 2017; NIH, 2023; Stasiłowicz et al., 2021). It took a long time to finally discover human cannabinoid receptors for in the 1980s; there are two subtypes: CB_1_ and CB_2_ (Kendall & Yudowski, 2017; Stasiłowicz et al., 2021; Svízenská, Dubový & Sulcová, 2008). Subsequently, the importance of the endocannabinoid system was clarified and endogenous ligands of the cannabinoid receptors—metabolites of arachidonic acid—were discovered. They include anandamide (AEA), 2-arachidonoylglycerol (2-AG), and palmitoylethanolamide. Less well-established endocannabinoids are virodhamin, noladin, and *N*-arachidonoyl dopamine (Mouslech & Valla, 2009). The main substances of marijuana or hashish are phytocannabinoids, *e.g*., Δ^9^-tetrahydrocannabinol (Δ^9^-THC, dronabinol, (-)-THC), Δ^8^-tetrahydrocannabinol (Δ^8^-THC), cannabidiol (CBD) and cannabinol (CBN). A total of almost 70 isolated cannabinoids are known (European Medicines Agency, 2020b; Gülck & Lindberg Møller, 2020). Currently, there is information on the therapeutic application of the above-mentioned herbal drugs, their extracts and/or individual active metabolites, as well as on some analogues with slight chemical structural modifications. Cannabis flos and Cannabis herba (European Medicines Agency, 2020b) of pharmaceutical quality (*Cannabis sativa* L., Cannabaceae) are used as analgesics in oncology and to treat neuropathic pain (oral use in the form of a water infusion, *i.e*., herbal tea, or as inhalation therapy) (Fitzcharles et al., 2021; Giacoppo, Bramanti & Mazzon, 2017; Hill et al., 2017; NIH, 2023; Stasiłowicz et al., 2021; Vulfsons et al., 2020). Synthetic agonists at cannabinoid receptors encompass dronabinol (Δ^9^-THC) and nabilone (both are used as antiemetic agents during cancer treatment and in the treatment of anorexia of patients with HIV) (Beal et al., 1995; DAC, 2001; Ng & Gupta, 2022). Cannabidiol (CBD) is used as an orphan drug in the therapy of specific forms of epilepsy: the Dravet syndrome (European Medicines Agency, 2014; Chen, Borgelt & Blackmer, 2019b) and the Lennox-Gastaut syndrome (European Medicines Agency, 2017c; Chen, Borgelt & Blackmer, 2019b). EMA granted an orphan designation for cannabidiol for the treatment of tuberous sclerosis (European Medicines Agency, 2018) and glioma (European Medicines Agency, 2016b). An inverse agonist at cannabinoid receptors, rimonabant, was tested in the treatment of addiction and obesity, but was withdrawn from the market due to side effects (2% incidence of depression) and suicidal risk (Bielawiec, Harasim-Symbor & Chabowski, 2020; Leite et al., 2009; European Pharmacopoeia, 2023h). Cannabis sativae oleum, the fatty oil obtained from Cannabis semen is important for the treatment of eczema and intertrigo (Sklenář, 2021).

Pharmacokinetic interactions occur mainly at the level of metabolism (Table 6). Phytocannabinoids are extensively metabolized by the cytochrome P_450_ (Czigle & Tóth, 2011, 2016). Cannabinoids inhibit CYP2C9 and CYP3A4 (Czigle & Tóth, 2011; Williamson, Driver & Baxter, 2009, 2013, 2018), while marijuana smoking induces CYP1A2 (Czigle & Tóth, 2011, 2016; Williamson, Driver & Baxter, 2009, 2013, 2018). THC and CBD inhibit CYP2D6, CYP2C19, CYP2B6 and CYP2J2 (Qian, Gurley & Markowitz, 2019). CYP3A4/5/7 is potentially inhibited by CBD (Qian, Gurley & Markowitz, 2019). Δ^9^-THC induces CYP1A1 also activates CYP2C9 (Czigle & Tóth, 2011, 2016; Qian, Gurley & Markowitz, 2019; Williamson, Driver & Baxter, 2009, 2013, 2018), the cannabinoids mixture also induces the expression of CYP2E1 (Czigle & Tóth, 2011, 2016; Williamson, Driver & Baxter, 2013) and CYP2D6 (Czigle & Tóth, 2011, 2016; Williamson, Driver & Baxter, 2009, 2013, 2018). UGT1A9 is inhibited by CBN and CBD, UGT2B7 is activated by CBN but inhibited by CBD (Qian, Gurley & Markowitz, 2019). Carboxylesterase 1 (CES1) is potentially inhibited by CBD and THC (Qian, Gurley & Markowitz, 2019). Pharmacodynamic interactions are related to the mechanism of action of cannabinoids (Table 6).

**Table 6:** Drug interactions of cannabinoids-containing herbal drugs (Cannabis flos, Cannabis herba, Cannabis resina).

ATC therapeutic subgroup	ATC pharmacological/chemical subgroup	Drugs	Pharmacokinetic basis of interaction	Pharmacodynamic basis of interaction	Manifestation of interaction	
Stomatological preparation, in dentistry (A01)	Salicylic acid and derivatives	Acetylsalicylic acid	CYP1A2 CYP2C9 CYP3A4		**↓** analgetic effect	Petersen, Bergien & Staerk (2021)
Antithrombotic agents (B01)						
Analgetics (N02)						
Antihypertensives (C02)	Imidazoline receptor agonists	Clonidine	CYP1A2	*Cannabis* has β-agonistic effect.	**↑** adverse reaction (tachycardia) **!** exclude marijuana	Cone, Welch & Lange (1988)
Analgesics (N02)	Other antimigraine preparations					
Ophtalmologicals (S01)	Sympathomimetics in glaucoma therapy					
Antivirals for systemic use (J05)	Protease inhibitors	Indinavir Nelfinavir	CYP3A4 P-gp		**↓** plasma level **↓** AUC (10%, and 14%)	Kosel et al. (2002)
Antineoplastic agents (L01)	Cytotoxic antibiotics and related substances	Bleomycin	CYP2B1	*Cannabis* and Δ^9^-THC are used as analgesics and antiemetics in oncology.	**↑** adverse reactions (cephalea, paresis, aphasia, stroke, exitus) **!** exclude marijuana	Merkle & Tavernier (2018)
	Platinum compounds	Cisplatin	CYP3A4	*Cannabis* and Δ^9^-THC are used as analgesics and antiemetics in oncology.	**↑** adverse reactions (cephalea, paresis, aphasia, stroke, exitus) **!** exclude marijuana **!** medicinal *Cannabis* herbal tea does not affect the level of cytostatics but inhalation (joint smoking) is risky	Marzęda et al. (2022)
	Topoisomerase 1 inhibitors	Irinotecan	CYP3A4	*Cannabis* and Δ^9^-THC are used as analgesics and antiemetics in oncology.	**↑** adverse reactions (cephalea, paresis, aphasia, stroke, exitus) **!** exclude marijuana **!** medicinal *Cannabis* herbal tea does not affect the level of cytostatics but inhalation (joint smoking) is risky	Engels et al. (2007)
Immunosuppressants drugs (L04)	Calcineurin inhibitors	Cyclosporine Tacrolimus	CYP3A4 P-gp		**↑** cyclosporine plasma level (73%–83%) **↓** metabolism	Czigle & Tóth (2011), Leino et al. (2019)
Anti-inflammatory and antirheumatic drugs (M01)	Anti-inflammatory and antirheumatic products, non-steroids	Celecoxib Diclofenac Ibuprofen Indometacin Naproxen Piroxicam	CYP2C9 CYP3A4	*Cannabis* affects prostaglandins levels.	antagonistic effect **↓** NSAIDs effect	Czigle & Tóth (2011), Emiga et al. (2020)
Analgesics (N02)	Opioids, natural opium alkaloids	Morphine Codeine Hydromorphone Oxymorphone	CYP2D6	Phytocannabinoids are CB_1_, CB_2_ receptor agonists.	**↑** Improvement in analgesic effects (without adverse reactions increase). **!** dosage adjustment (reduce the dose of anodyne by 60–100%)	Czigle & Tóth (2011), Fattore et al. (2004)
	Opioids, benzomorphan derivatives	Methadone	CYP3A4 CYP2C19	Phytocannabinoids are CB_1_, CB_2_ receptor agonists.	**↑** Improvement in analgesic effects (without adverse reactions increase). **!** dosage adjustment (reduce the dose of anodyne by 60–100%)	Madden, Tanco & Bruera (2020)
	Other analgesics and antipyretics, anilides	Paracetamol	CYP1A2 CYP3A4 CYP2E1	*Cannabis* affects prostaglandins levels.	**↓** analgetic effect	van Amerongen et al. (2018)
Antiepileptics (N03)	Antiepileptics, barbiturates and derivatives	Phenobarbital Pentobarbital Secobarbital	CYP2C9	Cannabinol is a CB_1_, CB_2_ receptor agonist, used as antiepileptic (Dravet-, and Lennox-Gastaut syndrome).	**↑** metabolism **↓** anticonvulsive effect	Czigle & Tóth (2011), Hollister (1986)
	Antiepileptics, hydantoin derivatives	Phenytoin	CYP2C9 CYP2C19	Cannabinol is a CB_1_, CB_2_ receptor agonist, used as antiepileptic (Dravet-, and Lennox-Gastaut syndrome).	**↑** metabolism **↓** anticonvulsive effect **!** exclude marijuana	Jessen (2004)
Psycholeptics (N05)	Antipsychotics	Chlorpromazine Clozapine Lithium	CYP1A2 CYP2D6	Phytocannabinoids are CB_1_, CB_2_ receptor agonists.	**↓** chlorpromazine plasma level **↑** chlorpromazine clearance (tobacco smoking: by 38%, joint smoking: by 50%, smoking both: by 107%) **!** exclude marijuana	Babatope et al. (2016), Brunette et al. (2011), Singh et al. (2020)
	Anxiolytics, benzodiazepine derivatives	Alprazolam Diazepam	CYP2C19 CYP3A4	Phytocannabinoids are CB_1_, CB_2_ receptor agonists.	**↓** anxiolytic effect	Lile, Kelly & Hays (2014)
	Hypnotics and sedatives, benzodiazepine derivatives	Midazolam	CYP2C19 CYP3A4	Phytocannabinoids are CB_1_, CB_2_ receptor agonists.	**↓** hypnotic effect	Twardowski, Link & Twardowski (2019)
Psychoanaleptics (N06)	Antidepressants, non-selective monoamine reuptake inhibitors	Amitriptyline Nortriptyline Desipramine Imipramine	CYP3A4 CYP2C9 CYP2D6 Pgp	*Cannabis* has β-agonistic effect.	additive β-agonistic effect **↑** adverse reactions (tachycardia, delirium) **!** exclude marijuana	Vázquez et al. (2020)
	Antidepressants, selective serotonin reuptake inhibitors	Fluoxetine Sertraline	CYP2D6 CYP3A4	Phytocannabinoids are CB_1_, CB_2_ receptor agonists.	synergistic (serotonergic) effect **↑** adverse reactions (serotonin syndrome, hypomanic periode risk) **!** exclude marijuana	Vaughn et al. (2021)
Other nervous system drugs (N07)	Drugs used in nicotine dependence	Nicotine (transdermally)	CYP2C9	Phytocannabinoids are CB_1_, CB_2_ receptor agonists.	additive effect **↑** adverse reactions (tachycardia, vigilantia) **!** exclude marijuana	Mohamed et al. (2011), Tucker et al. (2019)
	Drugs used in alcohol dependence	Disulfiram	CYP1A2 CYP3A4	Phytocannabinoids are CB_1_, CB_2_ receptor agonists.	**↑** adverse reactions (hypomanic syndrome) **!** exclude marijuana	Lacoursiere & Swatek (1983)
Drugs for obstructive airway diseases (R03)	Other systemic drugs for obstructive airway diseases, xanthines	Aminophylline Theophylline	CYP1A2	Cannabinoids have bronchodilatory effects.	**↑** metabolism **↓** plasma level **!** exclude marijuana	Antoniou, Bodkin & Ho (2020), Jusko et al. (1978)
All other therapeutic products (V03)	Nerve depressants	Ethanol	CYP2D6	Phytocannabinoids are CB_1_, CB_2_ receptor agonists.	additive effect **↑** adverse reaction (delirium) **↓** psychomotoric test results **!** exclude marijuana (**↑** fatal accident risk)	Mohamed et al. (2011)

**Note:**

ATC, Anatomical Therapeutic Chemical Classification System (WHO, 2023); ↑, increase (of); ↓, decrease (of); !, warning; CB_1_, cannabinoid receptor type 1; CB_2_, cannabinoid receptor type 2; ∆^9^-THC, ∆^9^-tetrahydrocannabinol; P-gp, P-glycoprotein.

## Preventing drug interactions/practical recommendations

### General:

- Patient compliance is essential (Czigle & Tóth, 2016).

- The medical doctor/pharmacist should be informed about self-medication (Czigle & Tóth, 2016).

- The duration of use of traditional herbal medicinal products (self-medication) should not exceed 3 months. If symptoms persist for more than 1 (or 2–3) weeks during the use of the traditional herbal medicinal product, a qualified physician or a qualified health care practitioner should be consulted (Czigle & Tóth, 2016; European Medicines Agency, 2023).

- For many (traditional) herbal medicinal products, use in children and adolescents has not been established due to the lack of adequate data (Czigle & Tóth, 2016; Williamson, Driver & Baxter, 2009, 2013, 2018).

- The elderly population is at risk due to reduced liver and kidney functions, on which drug clearance depends (Czigle & Tóth, 2016; Williamson, Driver & Baxter, 2009, 2013, 2018).

- In the absence of sufficient data, the use of herbal drugs during pregnancy and lactation is not recommended (Czigle & Tóth, 2016; Williamson, Driver & Baxter, 2009, 2013, 2018).

- Patients need to be monitored: for psychiatric, neurological (*e.g*., epilepsy), and/or cardiological diseases, for metabolic syndrome and diabetes mellitus (Czigle & Tóth, 2016).

- Patients using some medicines with high protein binding (*e.g*., warfarin) need to be monitored (Czigle & Tóth, 2016).

- Patients with any drugs that have a narrow therapeutic window or where it is necessary to keep serum levels at or above a suitable level (*e.g*., digoxin) should be more tightly observed (Czigle & Tóth, 2016).

- Hypersensitivity to the active substance(s) often occurs (Czigle & Tóth, 2016).

- If knowledge is available, the combination of substances that are inducers or inhibitors of cytochrome P_450_ isoenzymes (Czigle & Tóth, 2016; Zhao et al., 2021) and of the P-gp should be avoided (Czigle & Tóth, 2016).

- Many drugs and herbal medicines affect more than on type of target (receptor, enzyme *etc*.) (Czigle & Tóth, 2016; Williamson, Driver & Baxter, 2009, 2013, 2018).

- In the case of severe symptoms of interaction, it is usually the herbal medicine therapy that should be discontinued as first (Czigle & Tóth, 2016).

In addition to adhering to the general rules for preventing drug interactions, some more specific practical recommendations are summarized below for the herbal drugs listed in the previous chapters based on their pharmacodynamic properties and known adverse effects (Czigle & Tóth, 2016).

### Specific:

**Caffeine and herbal drugs containing caffeine**

- Drinking tea and/or coffee is not recommended before bedtime, as it can cause sleep disturbances due to the stimulating effect of caffeine (Czigle & Tóth, 2010b).

- Gastric and duodenal ulcers, cardiovascular disorders (*e.g*., hypertension and arrhythmia) and hyperthyroidism should be considered a contraindication during the use of herbal substances containing caffeine (green/black tea—Camelliae folium (non) fermentatum, coffee—Coffeae semen, guarana—Guaranae semen/Paulliniae semen/Guarana, maté leaf—Mate folium, cola—Colae semen); this applies both to the use of medicines and food/beverages (Czigle & Tóth, 2010b, 2016).

- When taking Mate folium to achieve flushing of the urinary tract, the fluid intake has to be observed (at least 2 L of water should be consumed daily). Conditions in which reduced fluid intake is recommended, for example, obstruction of the urinary tract, represent a contraindication. If complaints or symptoms such as fever, dysuria, spasms or blood in the urine occur during the use of the medicinal product, a qualified physician or qualified health care practitioner should be consulted (European Pharmacopoeia, 2023j).

***Ginkgo biloba***

- Preparations containing *Ginkgo* leaf or standardized extracts (Ginkgonis folium, Ginkgonis extractum siccum raffinatum et quantificatum) might increase susceptibility to bleeding; the medicinal product should be discontinued as a precaution at least 3 to 4 days before surgery (Czigle & Tóth, 2016).

- In patients with a pathologically increased bleeding tendency (hemorrhagic diathesis) and concomitant anticoagulant and antiplatelet treatment, the *Ginkgo biloba* medicinal product should only be used after consultation with a medical doctor (Czigle & Tóth, 2016).

- When patients with epilepsy take *Ginkgo* preparations (medicines or dietary supplements), an onset of further seizures promoted by the herbal drug cannot be excluded (Czigle & Tóth, 2016).

- Dietary supplements (foods) do not have pharmaceutical quality; they are not suitable for therapy. Herbal medicinal products must maintain quality, efficacy, and safety (Czigle et al., 2018).

***Hypericum perforatum***

- Intense UV exposure should be avoided in the course of treatment with St. John’s wort—Hyperici herba and its standardized extracts, due to phototoxicity (hypericism) (Czigle & Tóth, 2016).

***Valeriana officinalis* and *Humulus lupulus***

- In the course of treatment with valerian root—Valerianae radix and/or hop strobile—Lupuli flos affected patients should not drive or operate machinery, as the herbal drugs and extracts may impair such abilities (Czigle & Tóth, 2016; European Medicines Agency, 2005, 2013, 2015a, 2015c, 2017b).

***Cannabis sativa***

- In many countries, the use, cultivation, and distribution of marijuana (Cannabis herba) and hashish (Cannabis resina) are restricted and/or regulated by laws on narcotic and psychotropic substances (Czigle & Tóth, 2011, 2016).

- The potential risk of *Cannabis*-drug interactions is not negligible in clinical practice (Czigle & Tóth, 2011, 2016).

- Healthcare professionals should actively inquire about the use of *Cannabis*-based drugs and provide warning advice about it (Czigle & Tóth, 2011, 2016).

- Hemp extracts are not recommended for patients under 18 years of age, even in countries where they are marketed as registered medicines (Czigle & Tóth, 2011).

- Latent mental illnesses can manifest through the use of cannabis-based drugs (Czigle & Tóth, 2011, 2016).

- The use during pregnancy and lactation is contraindicated, as cannabinoids have a teratogenic effect (Czigle & Tóth, 2011, 2016).

## Conclusions

The possibility of drug interactions between conventional drugs and herbal medicines (or herbal food supplements) is an emerging concern for therapeutic safety, as subsequent side effects and direct toxicities constitute a possible hidden threat to successful clinical therapy. Simultaneous use of herbal products and other synthetic drugs can modify, increase, or decrease the therapeutic effects of a drug; in addition, new side effects can arise. Plausible cases of herb-drug interactions include: herbal medicines or beverages containing caffeine reduce the activity of sedatives and increase the side effects caused by sympathomimetic drugs; the stimulant effect of caffeine can oppose the hypnotic effects of *Valeriana officinalis*; additive anxiolytic effects are possible when benzodiazepines are combined with *Valeriana officinalis* or *Humulus lupulus*; bleeding may occur when anticoagulants/antiplatelet drugs are combined with *Ginkgo biloba*; serotonin syndrome can threaten patients who combine *Hypericum perforatum* with serotonin-affecting medications such as serotonin reuptake inhibitors. For medicinal *Cannabis*, antagonistic effects were observed for combinations with some NSAIDs, as well as a potentiation of the antinociceptive effect of anodynes. Patient compliance is essential. The medical doctor and pharmacist should be informed about self-medication.

## List of abbreviations

A_1_ and A_2_ receptors: adenosine 1 and 2 receptors
α_2_-agonistic effect: alpha 2 adrenergic agonist
ABCB1: cassette sub-family B member 1
ADME: absorption, distribution, metabolism, elimination/excretion
AEA: anandamide
2-AG: 2-arachidonoylglycerol
ATC system: Anatomical Therapeutic Chemical Classification System
CB_1_, CB_2_: cannabinoid receptors
CBD: cannabidiol
CBN: cannabinol
CES1: carboxylesterase 1
C_max_: maximum drug concentration
CNS: central nervous system
CYP1A1, CYP1A2, CYP2C8, CYP2B6, CYP2C9, CYP2C19, CYP2D6, CYP2E1, CYP2J2, CYP3A4, CYP3A4/5/7: cytochrome P450 isoenzymes
D_3_ receptor: dopamine receptor subtype 3
EGb 761: refined and quantified dry *Ginkgo* leaf extract
ECG: (-)-epicatechin-3-*O*-gallate
EGCG: (-)-epigallocatechin-3-*O*-gallate
EMA-HMPC: European Medicines Agency—Committee on Herbal Medicinal Products
EU: European Union
excl.: excluding
GABA: γ-aminobutyric acid
GABA_A_R: γ-aminobutyric acid receptor subtype A
H: histamine
5-HT: 5-hydroxytryptamine (serotonin)
5-HT_1A_, 5-HT_1BD_, 5-HT_5A_: 5-hydroxytryptamine (serotonin) receptors 5-HT_1A_, 5-HT_1BD_, 5-HT_5A_ subtypes
HIV: human immunodeficiency virus
INR: international normalized ratio
MAO: monoamine oxidase
MAO-A and MAO-B: monoamine oxidase A and B
MDR1: multidrug resistance protein 1
MRP1: multidrug resistance-associated protein 1
mGlu I and II: metabotropic glutamate receptors I and II
MT_1_ and MT_2_: melatonin receptors 1 and 2
Na^+^/K^+^-ATPase: sodium- and potassium-activated adenosine 5′-triphosphatase
NE: norepinephrine
NSAIDs: non-steroidal anti-inflammatory drugs
OATPs: organic anion-transporting polypeptides
OCTs: organic cation transporters
PAF: platelet-activating factor
PDE1, PDE4, PDE5: phosphodiesterase inhibitor
P-gp: P-glycoprotein
PT: prothrombin time
RCVS: reversible cerebral vasoconstriction syndrome
RYR: ryanodine receptor
Δ^8^-THC: Δ^8^-tetrahydrocannabinol
Δ^9^-THC: Δ^9^-tetrahydrocannabinol, dronabinol, (-)-*trans*-THC
TRPC1, TRPC3, TRPC4, TRPC5, TRPC6 and TRPC7: transient receptor potential cation channels
UGT: UDP-glucuronyltransferases
UGT1A9, UGT2B7: UDP-glucuronosyltransferase (UGT) 1A9 and 2B7
UV: ultraviolet
WHO: World Health Organization
↑: increase (of)
↓: decrease (of)
!: warning

## Additional Information and Declarations

### Data Availability

The following information was supplied regarding data availability: This is a literature review and did not utilize raw data.

## References

[ref-1] Abourashed EA, Koetter U, Brattström A (2004). *In vitro* binding experiments with a Valerian, hops and their fixed combination extract (Ze91019) to selected central nervous system receptors. Phytomedicine.

[ref-2] Ahlemeyer B, Krieglstein J (2003). Neuroprotective effects of *Ginkgo biloba* extract. Cellular and Molecular Life Sciences.

[ref-3] Alberti TB, Barbosa WL, Vieira JL, Raposo NR, Dutra RC (2017). (-)-β-Caryophyllene, a CB2 receptor-selective phytocannabinoid, suppresses motor paralysis and neuroinflammation in a murine model of multiple sclerosis. International Journal of Molecular Sciences.

[ref-4] Ali Y, Shams T, Wang K, Cheng Z, Li Y, Shu W, Bao X, Zhu L, Murray M, Zhou F (2020). The involvement of human organic anion transporting polypeptides (OATPs) in drug-herb/food interactions. Chinese Medicine.

[ref-5] Alscher DM, Klotz U (2003). Drug interaction of herbal tea containing St. John’s wort with cyclosporine. Transplant International.

[ref-6] Amaeze O, Eng H, Horlbogen L, Varma MVS, Slitt A (2021). Cytochrome P450 enzyme inhibition and herb-drug interaction potential of medicinal plant extracts used for management of diabetes in Nigeria. European Journal of Drug Metabolism and Pharmacokinetics.

[ref-7] Amjad W, Qureshi W, Farooq A, Sohail U, Khatoon S, Pervaiz S, Narra P, Hasan SM, Ali F, Ullah A, Guttmann S (2017). Gastrointestinal side effects of antiarrhythmic medications: a review of current literature. Cureus.

[ref-8] Andersson T, Holmberg J, Röhss K, Walan A (1998). Pharmacokinetics and effect on caffeine metabolism of the proton pump inhibitors, omeprazole, lansoprazole, and pantoprazole. British Journal of Clinical Pharmacology.

[ref-9] Anthony M, Romero K, Malone DC, Hines LE, Higgins L, Woosley RL (2009). Warfarin interactions with substances listed in drug information compendia and in the FDA-approved label for warfarin sodium. Clinical Pharmacology and Therapeutics.

[ref-10] Antoniou T, Bodkin J, Ho JM (2020). Drug interactions with cannabinoids. Canadian Medical Association Journal.

[ref-11] Astrup A, Breum L, Toubro S, Hein P, Quaade F (1992). The effect and safety of an ephedrine/caffeine compound compared to ephedrine, caffeine and placebo in obese subjects on an energy restricted diet. A double blind trial. International Journal of Obesity and Related Metabolic Disorders.

[ref-12] Babatope T, Chotalia J, Elkhatib R, Mohite S, Shah J, Goddu S, Patel RA, Aimienwanu OR, Patel D, Makanjuola T, Okusaga OO (2016). A study of the impact of cannabis on doses of discharge antipsychotic medication in individuals with schizophrenia or schizoaffective disorder. Psychiatry Journal.

[ref-13] Banin RM, Hirata BK, Andrade IS, Zemdegs JC, Clemente AP, Dornellas AP, Boldarine VT, Estadella D, Albuquerque KT, Oyama LM, Ribeiro EB, Telles MM (2014). Beneficial effects of *Ginkgo biloba* extract on insulin signaling cascade, dyslipidemia, and body adiposity of diet-induced obese rats. Brazilian Journal of Medical and Biological Research.

[ref-14] Batéjat D, Coste O, Van Beers P, Lagarde D, Piérard C, Beaumont M (2006). Prior sleep with zolpidem enhances the effect of caffeine or modafinil during 18 hours continuous work. Aviation, Space, and Environmental Medicine.

[ref-15] Beach CA, Mays DC, Guiler RC, Jacober CH, Gerber N (1986). Inhibition of elimination of caffeine by disulfiram in normal subjects and recovering alcoholics. Clinical Pharmacology & Therapeutics.

[ref-16] Beal JE, Olson R, Laubenstein L, Morales JO, Bellman P, Yangco B, Lefkowitz L, Plasse TF, Shepard KV (1995). Dronabinol as a treatment for anorexia associated with weight loss in patients with AIDS. Journal of Pain and Symptom Management.

[ref-17] Benkherouf AY, Eerola K, Soini SL, Uusi-Oukari M (2020). Humulone modulation of GABAA receptors and its role in hops sleep-promoting activity. Frontiers in Neuroscience.

[ref-18] Benowitz NL, Jacob P, Mayan H, Denaro C (1995). Sympathomimetic effects of paraxanthine and caffeine in humans. Clinical Pharmacology & Therapeutics.

[ref-19] Bensky D, Gamble A, Bensky LL (1986). Chinese Herbal Medicine Materia Medica.

[ref-20] Bielawiec P, Harasim-Symbor E, Chabowski A (2020). Phytocannabinoids: useful drugs for the treatment of obesity? Special focus on cannabidiol. Frontiers in Endocrinology.

[ref-21] Birnbaum GD, Birnbaum I, Ye Y, Birnbaum Y (2015). Statin-induced cardioprotection against ischemia-reperfusion injury: potential drug-drug interactions. Lesson to be learnt by translating results from animal models to the clinical settings. Cardiovascular Drugs and Therapy.

[ref-22] Bogacz A, Mrozikiewicz PM, Karasiewicz M, Bartkowiak-Wieczorek J, Majchrzycki M, Mikolajczak PL, Ozarowski M, Grzeskowiak E (2014). The influence of standardized Valeriana officinalis extract on the CYP3A1 gene expression by nuclear receptors in in vivo model. BioMed Research International.

[ref-23] Boiy A, Roelandts R, de Witte PA (2011). Photodynamic therapy using topically applied hypericin: comparative effect with methyl-aminolevulinic acid on UV induced skin tumours. Journal of Photochemistry and Photobiology B.

[ref-24] Bonetto N, Santelli L, Battistin L, Cagnin A (2007). Serotonin syndrome and rhabdomyolysis induced by concomitant use of triptans, fluoxetine and *Hypericum*. Cephalalgia.

[ref-25] Borrelli F, Izzo AA (2009). Herb-drug interactions with St John’s wort (*Hypericum perforatum*): an update on clinical observations. Journal of the American Association of Pharmaceutical Scientists.

[ref-26] Boswell-Smith V, Spina D, Page CP (2006). Phosphodiesterase inhibitors. British Journal of Pharmacology.

[ref-27] Brattström A (2007). Scientific evidence for a fixed extract combination (Ze 91019) from valerian and hops traditionally used as a sleep-inducing aid. Wiener Medizinische Wochenschrift.

[ref-28] Broughton LJ, Rogers HJ (1981). Decreased systemic clearance of caffeine due to cimetidine. British Journal of Clinical Pharmacology.

[ref-29] Brunette MF, Dawson R, O’Keefe CD, Narasimhan M, Noordsy DL, Wojcik J, Green AI (2011). A randomized trial of clozapine vs. other antipsychotics for cannabis use disorder in patients with schizophrenia. Journal of Dual Diagnosis.

[ref-30] Butterweck V (2003). Mechanism of action of St John’s wort in depression: what is known?. CNS Drugs.

[ref-31] Butterweck V, Brattstroem A, Grundmann O, Koetter U (2007). Hypothermic effects of hops are antagonized with the competitive melatonin receptor antagonist luzindole in mice. Journal of Pharmacy and Pharmacology.

[ref-32] Carbone K, Gervasi F (2022). An updated review of the genus humulus: a valuable source of bioactive compounds for health and disease prevention. Plants (Basel).

[ref-33] Carrillo JA, Benitez J (2000). Clinically significant pharmacokinetic interactions between dietary caffeine and medications. Clinical Pharmacokinetics.

[ref-34] Cauli O, Morelli M (2005). Caffeine and the dopaminergic system. Behavioural Pharmacology.

[ref-35] Cavadas C, Araújo I, Cotrim MD, Amaral T, Cunha AP, Macedo T, Ribeiro CF (1995). *In vitro* study on the interaction of Valeriana officinalis L. extracts and their amino acids on GABAA receptor in rat brain. Arzneimittelforschung.

[ref-36] Chen JW, Borgelt LM, Blackmer AB (2019b). Cannabidiol: a new hope for patients with Dravet or Lennox-Gastaut syndromes. Annals of Pharmacotherapy.

[ref-37] Chen TR, Wei LH, Guan XQ, Huang C, Liu ZY, Wang FJ, Hou J, Jin Q, Liu YF, Wen PH, Zhang SJ, Ge GB, Guo WZ (2019c). Biflavones from Ginkgo biloba as inhibitors of human thrombin. Bioorganic Chemistry.

[ref-38] Chen H, Zhou C, Yu M, Feng S, Ma Y, Liu Z, Zhang J, Ding T, Li B, Wang XT (2019a). The effect of Ginkgo biloba dropping pills on hemorheology and blood lipid: a systematic review of randomized trials. Evidence-Based Complementary and Alternative Medicine.

[ref-39] Chrościńska-Krawczyk M, Radzik I, Miziak B, Czuczwar SJ (2014). Safety considerations for patients with epilepsy taking antiepileptic drugs alongside caffeine or other methylxanthine derivatives. Expert Opinion on Drug Metabolism & Toxicology.

[ref-40] Cone EJ, Welch P, Lange WR (1988). Clonidine partially blocks the physiologic effects but not the subjective effects produced by smoking marijuana in male human subjects. Pharmacology Biochemistry and Behavior.

[ref-41] Cordier W, Steenkamp V (2011). Drug interactions in African herbal remedies. Drug Metabolism and Drug Interactions.

[ref-42] Cui YH, Zheng Y (2016). A meta-analysis on the efficacy and safety of St John’s wort extract in depression therapy in comparison with selective serotonin reuptake inhibitors in adults. Neuropsychiatric Disease and Treatment.

[ref-43] Czigle S, Tóth J (2009a). Interakcie liečiv a niektorých liečivých rastlín s extraktmi a obsahovými látkami ginka dvojlaločného (*Ginkgo biloba* L.). Liekové interakcie.

[ref-44] Czigle S, Tóth J (2009b). Interakcie liečiv a niektorých liečivých rastlín s extraktmi a ostatnými látkami ľubovníka bodkovaného (*Hypericum perforatum* L.). Liekové interakcie.

[ref-45] Czigle S, Tóth J (2010a). Interakcie liečiv a niektorých liečivých rastlín s extraktmi a obsahovými látkami valeriány lekárskej (*Valeriana officinalis* L.). Liekové interakcie.

[ref-46] Czigle S, Tóth J (2010b). Interakcie rastlinných psychostimulantov s obsahom kofeínu a metylxantínov s liečivami a niektorými liečivými rastlinami. Liekové interakcie.

[ref-47] Czigle S, Tóth J (2011). Interakcie konopy (*Cannabis* L.), jej živice a obsahových látok s liečivami a niektorými liečivými rastlinami. Liekové interakcie.

[ref-48] Czigle S, Tóth J (2016). Fytofarmaká a potraviny—klinicky významné interakcie pre všeobecných lekárov.

[ref-49] Czigle S, Tóth J, Jedlinszki N, Háznagy-Radnai E, Csupor D, Tekeľová D (2018). *Ginkgo biloba* food supplements on the European Market—adulteration patterns revealed by quality control of selected samples. Planta Medica.

[ref-50] DAC (2001). Deutscher Arzneimittel-Codex.

[ref-51] Del Valle-Mojica LM, Ortíz JG (2012). Anxiolytic properties of Valeriana officinalis in the zebrafish: a possible role for metabotropic glutamate receptors. Planta Medica.

[ref-52] Dhiman P, Malik N, Sobarzo-Sánchez E, Uriarte E, Khatkar A (2019). Quercetin and related chromenone derivatives as monoamine oxidase inhibitors: targeting neurological and mental disorders. Molecules.

[ref-53] Diener HC, Pfaffenrath V, Pageler L, Peil H, Aicher B (2005). The fixed combination of acetylsalicylic acid, paracetamol and caffeine is more effective than single substances and dual combination for the treatment of headache: a multicentre, randomized, double-blind, single-dose, placebo-controlled parallel group study. Cephalalgia.

[ref-54] Dietz BM, Chen SN, Alvarenga RFR, Dong H, Nikolić D, Biendl M, van Breemen RB, Bolton JL, Pauli GF (2017). DESIGNER extracts as tools to balance estrogenic and chemopreventive activities of botanicals for women’s health. Journal of Natural Products.

[ref-55] Dietz BM, Mahady GB, Pauli GF, Farnsworth NR (2005). Valerian extract and valerenic acid are partial agonists of the 5-HT5a receptor *in vitro*. Brain Research Molecular Brain Research.

[ref-56] Drugs (2023). Drugs.com. https://www.drugs.com/monograph/pentazocine.html.

[ref-57] Dürr D, Stieger B, Kullak-Ublick GA, Rentsch KM, Steinert HC, Meier PJ, Fattinger K (2000). St John’s Wort induces intestinal P-glycoprotein/MDR1 and intestinal and hepatic CYP3A4. Clinical Pharmacology & Therapeutics.

[ref-58] Eggertsen R, Andreasson A, Andrén L (2007). Effects of treatment with a commercially available St John’s Wort product (Movina) on cholesterol levels in patients with hypercholesterolemia treated with simvastatin. Scandinavian Journal of Primary Health Care.

[ref-86] Emiga M, Kafaiea J, Onga S, Lia X (2020). Cannabidiol and non-steroidal anti-inflammatory drug interactions: a case of drug-induced aseptic meningitis. Journal of Neurology Research.

[ref-87] Engels FK, de Jong FA, Sparreboom A, Mathot RA, Loos WJ, Kitzen JJ, de Bruijn P, Verweij J, Mathijssen RH (2007). Medicinal cannabis does not influence the clinical pharmacokinetics of irinotecan and docetaxel. The Oncologist.

[ref-59] European Medicines Agency (2005). Assessment report on *Humulus lupulus* L., flos.

[ref-60] European Medicines Agency (2008a). Assessment report on *Ilex paraguariensis* St. Hilaire, folium.

[ref-61] European Medicines Agency (2008b). Community herbal monograph on *Ilex paraguariensis* St. Hilaire, folium.

[ref-62] European Medicines Agency (2010a). Assessment report on *Cola nitida* (Vent.) Schott et Endl. and its varieties and *Cola acuminata* (P. Beauv.) Schott et Endl., semen.

[ref-63] European Medicines Agency (2010b). Community herbal monograph on *Cola nitida* (Vent.) Schott et Endl. and its varieties and *Cola acuminata* (P. Beauv.) Schott et Endl., semen.

[ref-64] European Medicines Agency (2011a). Assessment report on *Paullinia cupana* Kunth ex H.B.K. var. *sorbilis* (Mart.) Ducke, semen.

[ref-65] European Medicines Agency (2011b). Community herbal monograph on *Paullinia cupana* Kunth ex H.B.K. var. *sorbilis* (Mart.) Ducke, semen.

[ref-66] European Medicines Agency (2012a). Assessment report on *Camellia sinensis* (L.) Kuntze, non fermentatum folium.

[ref-67] European Medicines Agency (2012b). Assessment report on *Ginkgo biloba* L., folium.

[ref-68] European Medicines Agency (2012c). Community herbal monograph on *Camellia sinensis* (L.) Kuntze, non fermentatum folium.

[ref-69] European Medicines Agency (2012d). European Union herbal monograph on *Ginkgo biloba* L., folium.

[ref-70] European Medicines Agency (2013). European Union herbal monograph on *Humulus lupulus* L., flos.

[ref-71] European Medicines Agency (2014). Public summary of opinion on orphan designation. Cannabidiol for the treatment of Dravet syndrome.

[ref-72] European Medicines Agency (2015a). Assessment report on *Valeriana officinalis* L., radix and *Valeriana officinalis* L., aetheroleum.

[ref-73] European Medicines Agency (2015b). European Union herbal monograph on *Valeriana officinalis* L., aetheroleum.

[ref-74] European Medicines Agency (2015c). European Union herbal monograph on *Valeriana officinalis* L., radix.

[ref-75] European Medicines Agency (2016a). Assessment report on *Hypericum perforatum* L., herba.

[ref-76] European Medicines Agency (2016b). Public summary of opinion on orphan designation. Delta-9-tetrahydrocannabinol and cannabidiol from extracts of the *Cannabis sativa* L. plant for the treatment glioma.

[ref-77] European Medicines Agency (2017a). European Union herbal monograph on Species sedativae.

[ref-78] European Medicines Agency (2017b). European Union herbal monograph on *Valeriana officinalis* L., radix and *Humulus lupulus* L., flos.

[ref-79] European Medicines Agency (2017c). Public summary of opinion on orphan designation. Cannabidiol for the treatment of Lennox-Gastaut syndrome.

[ref-80] European Medicines Agency (2018). Public summary of opinion on orphan designation. Cannabidiol for the treatment of tuberous sclerosis.

[ref-81] European Medicines Agency (2020a). Addendum to Assessment report on *Ilex paraguariensis* St. Hilaire, folium.

[ref-82] European Medicines Agency (2020b). Compilation of terms and definitions for Cannabis-derived medicinal products.

[ref-83] European Medicines Agency (2021). European Union herbal monograph on *Hypericum perforatum* L., herba.

[ref-84] European Medicines Agency (2022). Addendum to Assessment report on *Camellia sinensis* (L.) Kuntze, non fermentatum folium.

[ref-85] European Medicines Agency (2023). Herbal medicines. https://www.ema.europa.eu/en/medicines/field_ema_web_categories%253Aname_field/Herbal/field_ema_herb_outcome/european-union-herbal-monograph-254.

[ref-182] European Pharmacopoeia (2023a). Cola. European Pharmacopoeia.

[ref-183] European Pharmacopoeia (2023b). Ginkgo dry extract, refined and quantified. European Pharmacopoeia.

[ref-184] European Pharmacopoeia (2023c). Ginkgo leaf. European Pharmacopoeia.

[ref-185] European Pharmacopoeia (2023d). Green tea. European Pharmacopoeia.

[ref-186] European Pharmacopoeia (2023e). Guarana. European Pharmacopoeia.

[ref-187] European Pharmacopoeia (2023f). Herbal drug extracts. European Pharmacopoeia.

[ref-188] European Pharmacopoeia (2023g). Herbal drug preparations. European Pharmacopoeia.

[ref-189] European Pharmacopoeia (2023h). Herbal drugs. European Pharmacopoeia.

[ref-190] European Pharmacopoeia (2023i). Hop strobile. European Pharmacopoeia.

[ref-191] European Pharmacopoeia (2023j). Mate leaf. European Pharmacopoeia.

[ref-192] European Pharmacopoeia (2023k). St. John’s wort dry extract, quantified. European Pharmacopoeia.

[ref-193] European Pharmacopoeia (2023l). St. John’s wort. European Pharmacopoeia.

[ref-194] European Pharmacopoeia (2023m). Valerian dry aqueous extract. European Pharmacopoeia.

[ref-195] European Pharmacopoeia (2023n). Valerian dry hydroalcoholic extract. European Pharmacopoeia.

[ref-196] European Pharmacopoeia (2023o). Valerian root. European Pharmacopoeia.

[ref-197] European Pharmacopoeia (2023p). Valerian root, cut. European Pharmacopoeia.

[ref-198] European Pharmacopoeia (2023q). Valerian tincture. European Pharmacopoeia.

[ref-88] Faber MS, Jetter A, Fuhr U (2005). Assessment of CYP1A2 activity in clinical practice: why, how, and when?. Basic & Clinical Pharmacology & Toxicology.

[ref-89] Fantoli U (1981). Caffeina ed anticoncezionali orali [Caffeine and oral contraceptives]. Recenti Progressi in Medicina.

[ref-90] Fattore L, Cossu G, Spano MS, Deiana S, Fadda P, Scherma M, Fratta W (2004). Cannabinoids and reward: interactions with the opioid system. Critical Reviews in Neurobiology.

[ref-91] Feinberg I, Schoepp DD, Hsieh KC, Darchia N, Campbell IG (2005). The metabotropic glutamate (mGLU)2/3 receptor antagonist LY341495 [2S-2-amino-2-(1S,2S-2-carboxycyclopropyl-1-yl)-3-(xanth-9-yl)propanoic acid] stimulates waking and fast electroencephalogram power and blocks the effects of the mGLU2/3 receptor agonist ly379268 [(-)-2-oxa-4-aminobicyclo[3.1.0]hexane-4,6-dicarboxylate] in rats. Journal of Pharmacology and Experimental Therapeutic.

[ref-92] Fitzcharles MA, Petzke F, Tölle TR, Häuser W (2021). Cannabis-based medicines and medical cannabis in the treatment of nociplastic pain. Drugs.

[ref-93] Foster BC, Arnason JT, Saleem A, Tam TW, Liu R, Mao J, Desjardins S (2011). Comparative study of hops-containing products on human cytochrome P450-mediated metabolism. Journal of Agricultural and Food Chemistry.

[ref-94] Franco L, Sánchez C, Bravo R, Rodríguez AB, Barriga C, Romero E, Cubero J (2012). The sedative effect of non-alcoholic beer in healthy female nurses. PLOS ONE.

[ref-95] Fuzzati N, Pace R, Villa F (2003). A simple HPLC-UV method for the assay of ginkgolic acids in *Ginkgo biloba* extracts. Fitoterapia.

[ref-96] Giacoppo S, Bramanti P, Mazzon E (2017). Sativex in the management of multiple sclerosis-related spasticity: an overview of the last decade of clinical evaluation. Multiple Sclerosis and Related Disorders.

[ref-97] Granados-Soto V, Castañeda-Hernández G (1999). A review of the pharmacokinetic and pharmacodynamic factors in the potentiation of the antinociceptive effect of nonsteroidal anti-inflammatory drugs by caffeine. Journal of Pharmacological and Toxicological Methods.

[ref-98] Grundmann O, Brattström A, Koetter U, Butterweck V (2006). Hypothermic effects of hops could be antagonized with the competitive melatonin receptor antagonist luzindole. Planta Medica.

[ref-100] Gülck T, Lindberg Møller B (2020). Phytocannabinoids: origins and biosynthesis. Trends in Plant Science.

[ref-99] Guo J, Zhu X, Badawy S, Ihsan A, Liu Z, Xie C, Wang X (2021). Metabolism and mechanism of human cytochrome P450 enzyme 1A2. Current Drug Metabolism.

[ref-116] Härtter S, Korhonen T, Lundgren S, Rane A, Tolonen A, Turpeinen M, Laine K (2006). Effect of caffeine intake 12 or 24 hours prior to melatonin intake and CYP1A2*1F polymorphism on CYP1A2 phenotyping by melatonin. Basic & Clinical Pharmacology & Toxicology.

[ref-101] Hall SD, Wang Z, Huang SM, Hamman MA, Vasavada N, Adigun AQ, Hilligoss JK, Miller M, Gorski JC (2003). The interaction between St John’s wort and an oral contraceptive. Clinical Pharmacology & Therapeutics.

[ref-102] Hanrahan JR, Chebib M, Johnston GA (2011). Flavonoid modulation of GABA(A) receptors. British Journal of Pharmacology.

[ref-103] Hansten PD (2018). The underrated risks of tamoxifen drug interactions. European Journal of Drug Metabolism and Pharmacokinetics.

[ref-104] Harpsøe NG, Andersen LP, Gögenur I, Rosenberg J (2015). Clinical pharmacokinetics of melatonin: a systematic review. European Journal of Clinical Pharmacology.

[ref-105] Harrigan M, McGowan C, Hood A, Ferrucci LM, Nguyen T, Cartmel B, Li FY, Irwin ML, Sanft T (2021). Dietary supplement use and interactions with tamoxifen and aromatase inhibitors in breast cancer survivors enrolled in lifestyle interventions. Nutrients.

[ref-106] Hasler A, Gross GA, Meier B, Sticher O (1992). Complex flavonol glycosides of the leaves of *Ginkgo biloba*. Phytochemistry.

[ref-107] Hasler A, Meier B (1993). *Ginkgo biloba*—content of flavonoids and terpenes from leaves during the harvest time and from full extracts determined by chromatographic and biological methods. Planta Medica.

[ref-108] Hebert MF, Park JM, Chen YL, Akhtar S, Larson AM (2004). Effects of St. John’s wort (*Hypericum perforatum*) on tacrolimus pharmacokinetics in healthy volunteers. The Journal of Clinical Pharmacology.

[ref-109] Hellum BH, Nilsen OG (2008). *In vitro* inhibition of CYP3A4 metabolism and P-glycoprotein-mediated transport by trade herbal products. Basic & Clinical Pharmacology & Toxicology.

[ref-110] Hemachandra LP, Madhubhani P, Chandrasena R, Esala P, Chen SN, Main M, Lankin DC, Scism RA, Dietz BM, Pauli GF, Thatcher GR, Bolton JL (2012). Hops (Humulus lupulus) inhibits oxidative estrogen metabolism and estrogen-induced malignant transformation in human mammary epithelial cells (MCF-10A). Cancer Prevention Research.

[ref-111] Hibino G, Moritani T, Kawada T, Fushiki T (1997). Caffeine enhances modulation of parasympathetic nerve activity in humans: quantification using power spectral analysis. The Journal of Nutrition.

[ref-112] Hill KP, Palastro MD, Johnson B, Ditre JW (2017). Cannabis and pain: a clinical review. Cannabis and Cannabinoid Research.

[ref-113] Hollister LE (1986). Interactions of cannabis with other drugs in man. NIDA Research Monograph.

[ref-114] Horvat O, Raskovic A, Jakovljevic V, Sabo J, Berenji J (2007). Interaction of alcoholic extracts of hops with cocaine and paracetamol in mice. European Journal of Drug Metabolism and Pharmacokinetics.

[ref-115] Hwang HS, Baldo MP, Rodriguez JP, Faggioni M, Knollmann BC (2019). Efficacy of flecainide in catecholaminergic polymorphic ventricular tachycardia is mutation-independent but reduced by calcium overload. Frontiers in Physiology.

[ref-117] Isokawa M (2016). Caffeine-induced suppression of GABAergic inhibition and calcium-independent metaplasticity. Neural Plasticity.

[ref-118] Ivic L, Sands TT, Fishkin N, Nakanishi K, Kriegstein AR, Strømgaard K (2003). Terpene trilactones from *Ginkgo biloba* are antagonists of cortical glycine and GABA(A) receptors. Journal of Biological Chemistry.

[ref-119] Jakovljevic V, Popovic M, Raskovic A, Sabo A, Horvat O, Mitic R, Vasic R (2009). Effect of aroma and magnum hops extracts and paracetamol on antioxidant liver parameters in mice. European Journal of Drug Metabolism and Pharmacokinetics.

[ref-120] James JS (2000). St. John’s wort warning: do not combine with protease inhibitors, NNRTIs. AIDS Treatment News.

[ref-121] Jankiewicz K, Chrościńska-Krawczyk M, Błaszczyk B, Czuczwar SJ (2007). Kofeina a leki przeciwpadaczkowe: dane doświadczalne i kliniczne [Caffeine and antiepileptic drugs: experimental and clinical data]. Przeglad Lekarski.

[ref-122] Jayaraj RL, Beiram R, Azimullah S, Mf NM, Ojha SK, Adem A, Jalal FY (2020). Valeric acid protects dopaminergic neurons by suppressing oxidative stress, neuroinflammation and modulating autophagy pathways. International Journal of Molecular Sciences.

[ref-123] Jessen K (2004). Recreational use of phenytoin, marijuana, and alcohol: a case report. Neurology.

[ref-124] Jiang Y, Zhou Y, Song S, Fan S, Gao Y, Li Y, Huang M, Bi H (2022). St. John’s wort exacerbates acetaminophen-induced liver injury by activation of PXR and CYP-mediated bioactivation. Toxicological Sciences.

[ref-125] Johnston GA, Hanrahan JR, Chebib M, Duke RK, Mewett KN (2006). Modulation of ionotropic GABA receptors by natural products of plant origin. Advances in Pharmacology.

[ref-126] Jones G (2008). Caffeine and other sympathomimetic stimulants: modes of action and effects on sports performance. Essays in Biochemistry.

[ref-127] Jung HY, Yoo DY, Nam SM, Kim JW, Choi JH, Yoo M, Lee S, Yoon YS, Hwang IK (2015). Valerenic acid protects against physical and psychological stress by reducing the turnover of serotonin and norepinephrine in mouse hippocampus-amygdala region. Journal of Medicinal Food.

[ref-128] Jusko WJ, Schentag JJ, Clark JH, Gardner M, Yurchak AM (1978). Enhanced biotransformation of theophylline in marihuana and tobacco smokers. Clinical Pharmacology & Therapeutics.

[ref-129] Ke J, Li MT, Huo YJ, Cheng YQ, Guo SF, Wu Y, Zhang L, Ma J, Liu AJ, Han Y (2021). The synergistic effect of Ginkgo biloba extract 50 and aspirin against platelet aggregation. Drug Design, Development and Therapy.

[ref-130] Kelber O, Nieber K, Kraft K (2014). Valerian: no evidence for clinically relevant interactions. Evidence-Based Complementary and Alternative Medicine.

[ref-131] Kendall DA, Yudowski GA (2017). Cannabinoid receptors in the central nervous system: their signaling and roles in disease. Frontiers in Cellular Neuroscience.

[ref-132] Khom S, Baburin I, Timin E, Hohaus A, Trauner G, Kopp B, Hering S (2007). Valerenic acid potentiates and inhibits GABA(A) receptors: molecular mechanism and subunit specificity. Neuropharmacology.

[ref-133] Klauke AL, Racz I, Pradier B, Markert A, Zimmer AM, Gertsch J, Zimmer A (2014). The cannabinoid CB_2_ receptor-selective phytocannabinoid beta-caryophyllene exerts analgesic effects in mouse models of inflammatory and neuropathic pain. European Neuropsychopharmacology.

[ref-134] Kleber E, Obry T, Hippeli S, Schneider W, Elstner EF (1999). Biochemical activities of extracts from *Hypericum perforatum* L. 1st communication: inhibition of dopamine-beta-hydroxylase. Arzneimittelforschung.

[ref-135] Kong H, Jones PP, Koop A, Zhang L, Duff HJ, Chen SR (2008). Caffeine induces Ca2+ release by reducing the threshold for luminal Ca2+ activation of the ryanodine receptor. Biochemical Journal.

[ref-136] Kosel BW, Aweeka FT, Benowitz NL, Shade SB, Hilton JF, Lizak PS, Abrams DI (2002). The effects of cannabinoids on the pharmacokinetics of indinavir and nelfinavir. AIDS.

[ref-137] Kot M, Daniel WA (2008). Caffeine as a marker substrate for testing cytochrome P450 activity in human and rat. Pharmacological Reports.

[ref-138] Kroon LA (2007). Drug interactions with smoking. American Journal of Health-System Pharmacy.

[ref-139] Kubota Y, Tanaka N, Kagota S, Nakamura K, Kunitomo M, Umegaki K, Shinozuka K (2006). Effects of Ginkgo biloba extract on blood pressure and vascular endothelial response by acetylcholine in spontaneously hypertensive rats. Journal of Pharmacy and Pharmacology.

[ref-140] Lacoursiere RB, Swatek R (1983). Adverse interaction between disulfiram and marijuana: a case report. American Journal of Psychiatry.

[ref-141] Ladner DP, Klein SD, Steiner RA, Walt H (2001). Synergistic toxicity of delta-aminolaevulinic acid-induced protoporphyrin IX used for photodiagnosis and *Hypericum* extract, a herbal antidepressant. British Journal of Dermatology.

[ref-142] Lau WC, Welch TD, Shields T, Rubenfire M, Tantry US, Gurbel PA (2011). The effect of St John’s Wort on the pharmacodynamic response of clopidogrel in hyporesponsive volunteers and patients: increased platelet inhibition by enhancement of CYP3A4 metabolic activity. Journal of Cardiovascular Pharmacology.

[ref-143] Leino AD, Emoto C, Fukuda T, Privitera M, Vinks AA, Alloway RR (2019). Evidence of a clinically significant drug-drug interaction between cannabidiol and tacrolimus. American Journal of Transplantation.

[ref-144] Leistner E, Drewke C (2010). *Ginkgo biloba* and ginkgotoxin. Journal of Natural Products.

[ref-145] Leite CE, Mocelin CA, Petersen GO, Leal MB, Thiesen FV (2009). Rimonabant: an antagonist drug of the endocannabinoid system for the treatment of obesity. Pharmacological Reports.

[ref-146] Lile JA, Kelly TH, Hays LR (2014). Separate and combined effects of the GABAA positive allosteric modulator diazepam and Δ^9^-THC in humans discriminating Δ^9^-THC. Drug and Alcohol Dependence.

[ref-147] Loke WH, Hinrichs JV, Ghoneim MM (1985). Caffeine and diazepam: separate and combined effects on mood, memory, and psychomotor performance. Psychopharmacology.

[ref-148] Lopez F, Miller LG, Greenblatt DJ, Kaplan GB, Shader RI (1989). Interaction of caffeine with the GABAA receptor complex: alterations in receptor function but not ligand binding. European Journal of Pharmacology.

[ref-149] Lundahl A, Hedeland M, Bondesson U, Knutson L, Lennernäs HT (2009). The effect of St. John’s wort on the pharmacokinetics, metabolism and biliary excretion of finasteride and its metabolites in healthy men. European Journal of Pharmaceutical Sciences.

[ref-150] Madden K, Tanco K, Bruera E (2020). Clinically significant drug-drug interaction between methadone and cannabidiol. Pediatrics.

[ref-151] Manikandan P, Nagini S (2018). Cytochrome P450 structure, function and clinical significance: a review. Current Drug Targets.

[ref-152] Markowitz JS, DeVane CL, Boulton DW, Carson SW, Nahas Z, Risch SC (2000). Effect of St. John’s wort (*Hypericum perforatum*) on cytochrome P-450 2D6 and 3A4 activity in healthy volunteers. Life Sciences.

[ref-153] Marzęda P, Wróblewska-Łuczka P, Drozd M, Florek-Łuszczki M, Załuska-Ogryzek K, Łuszczki JJ (2022). Cannabidiol interacts antagonistically with cisplatin and additively with mitoxantrone in various melanoma cell lines—an isobolographic analysis. International Journal of Molecular Sciences.

[ref-154] Mathijssen RH, Verweij J, de Bruijn P, Loos WJ, Sparreboom A (2002). Effects of St. John’s wort on irinotecan metabolism. Journal of the National Cancer Institute.

[ref-155] Mauro VF, Mauro LS, Kleshinski JF, Khuder SA, Wang Y, Erhardt PW (2003). Impact of *Ginkgo biloba* on the pharmacokinetics of digoxin. American Journal of Therapeutics.

[ref-156] McClatchey WC, Mahady GB, Bennett BC, Shiels L, Savo V (2009). Ethnobotany as a pharmacological research tool and recent developments in CNS-active natural products from ethnobotanical sources. Pharmacology & Therapeutics.

[ref-157] Meier B, Hasler A, Sticher O (1992). HPLC analysis of the main *Ginkgo* acylflavonol glycosides. Planta Medica.

[ref-158] Merkle S, Tavernier SS (2018). *Cannabis* use and bleomycin: an overview and case study of pulmonary toxicity. Clinical Journal of Oncology Nursing.

[ref-159] Mohamed WM, Ben Hamida S, Cassel JC, de Vasconcelos AP, Jones BC (2011). MDMA: interactions with other psychoactive drugs. Pharmacology Biochemistry & Behavior.

[ref-160] Monteiro R, Becker H, Azevedo I, Calhau C (2006). Effect of hop (*Humulus lupulus* L.) flavonoids on aromatase (estrogen synthase) activity. Journal of Agricultural and Food Chemistry.

[ref-161] Mooiman KD, Maas-Bakker RF, Hendrikx JJ, Bank PC, Rosing H, Beijnen JH, Schellens JH, Meijerman IT (2014). The effect of complementary and alternative medicines on CYP3A4-mediated metabolism of three different substrates: 7-benzyloxy-4-trifluoromethyl-coumarin, midazolam and docetaxel. Journal of Pharmacy and Pharmacology.

[ref-162] Morimoto T, Kotegawa T, Tsutsumi K, Ohtani Y, Imai H, Nakano S (2004). Effect of St. John’s wort on the pharmacokinetics of theophylline in healthy volunteers. The Journal of Clinical Pharmacology.

[ref-163] Mouslech Z, Valla V (2009). Endocannabinoid system: an overview of its potential in current medical practice. Neuroendocrinology Letters.

[ref-165] Müller LG, Salles L, Lins HA, Feijó PR, Cassel E, Vargas R, von Poser GL, Noël F, Quintas LE, Rates SM (2015). Effects of diene valepotriates from *Valeriana glechomifolia* on Na^+^/K^+^-ATPase activity in the cortex and hippocampus of mice. Planta Medica.

[ref-164] Munn Z, Peters MDJ, Stern C, Tufanaru C, McArthur A, Aromataris E (2018). Systematic review or scoping review? Guidance for authors when choosing between a systematic or scoping review approach. BMC Medical Research Methodology.

[ref-166] Nagy M, Mučaji P, Grančai D (2017). Farmakognózia - biologicky aktívne rastlinné metabolity a ich zdroje.

[ref-167] Nehlig A (2018). Interindividual differences in caffeine metabolism and factors driving caffeine consumption. Pharmacological Reviews Pharmacological Reviews.

[ref-168] Newman DJ, Cragg GM (2020). Natural products as sources of new drugs over the nearly four decades from 01/1981 to 09/2019. Journal of Natural Products.

[ref-169] Ng JY (2021). Insight into the characteristics of research published in traditional, complementary, alternative, and integrative medicine journals: a bibliometric analysis. BMC Complementary Medicine and Therapies.

[ref-170] Ng T, Gupta V (2022). Tetrahydrocannabinol (THC).

[ref-171] Nicolussi S, Drewe J, Butterweck V, Meyer Zu Schwabedissen HE (2020). Clinical relevance of St. John’s wort drug interactions revisited. British Journal of Pharmacology.

[ref-172] NIH (2023). Clinical trials. https://www.clinicaltrials.gov/ct2/results/browse?cond=cannabis&brwse=cond_alpha_all.

[ref-173] Nomani H, Moghadam AT, Emami SA, Mohammadpour AH, Johnston TP, Sahebkar A (2019). Drug interactions of cola-containing drinks. Clinical Nutrition.

[ref-174] Oberpichler H, Sauer D, Rossberg C, Mennel HD, Krieglstein J (1990). PAF antagonist ginkgolide B reduces postischemic neuronal damage in rat brain hippocampus. Journal of Cerebral Blood Flow & Metabolism.

[ref-175] Orhan IE (2021). A review focused on molecular mechanisms of anxiolytic effect of *Valerina officinalis* L. in connection with its phytochemistry through *in vitro/in vivo* studies. Current Pharmaceutical Design.

[ref-176] Ortiz JG, Nieves-Natal J, Chavez P (1999). Effects of Valeriana officinalis extracts on [3H]flunitrazepam binding, synaptosomal [3H]GABA uptake, and hippocampal [3H]GABA release. Neurochemical Research.

[ref-177] Pandian BA, Sathishraj R, Djanaguiraman M, Prasad PVV, Jugulam M (2020). Role of cytochrome P450 enzymes in plant stress response. Antioxidants (Basel).

[ref-178] Park SH, Sim YB, Kang YJ, Kim SS, Kim CH, Kim SJ, Seo JY, Lim SM, Suh HW (2012). Hop extract produces antinociception by acting on opioid system in mice. Korean Journal of Physiology & Pharmacology.

[ref-179] Patwardhan RV, Desmond PV, Johnson RF, Schenker S (1980). Impaired elimination of caffeine by oral contraceptive steroids. Journal of Laboratory and Clinical Medicine.

[ref-180] Pentel P (1984). Toxicity of over-the-counter stimulants. The Journal of the American Medical Association.

[ref-181] Petersen MJ, Bergien SO, Staerk D (2021). A systematic review of possible interactions for herbal medicines and dietary supplements used concomitantly with disease-modifying or symptom-alleviating multiple sclerosis drugs. Phytotherapy Research.

[ref-199] Prely H, Herledan C, Caffin AG, Baudouin A, Larbre V, Maire M, Schwiertz V, Vantard N, Ranchon F, Rioufol C (2022). Real-life drug-drug and herb-drug interactions in outpatients taking oral anticancer drugs: comparison with databases. Journal of Cancer Research and Clinical Oncology.

[ref-200] Qian Y, Gurley BJ, Markowitz JS (2019). The potential for pharmacokinetic interactions between cannabis products and conventional medications. Journal of Clinical Psychopharmacology.

[ref-201] Ramassamy C, Christen Y, Clostre F, Costentin JT (1992). The Ginkgo biloba extract, EGb761, increases synaptosomal uptake of 5-hydroxytryptamine: in-vitro and ex-vivo studies. Journal of Pharmacy and Pharmacology.

[ref-202] Raskovic A, Horvat O, Jakovljevic V, Sabo J, Vasic R (2007). Interaction of alcoholic extracts of hops with pentobarbital and diazepam in mice. European Journal of Drug Metabolism and Pharmacokinetics.

[ref-203] Raskovic A, Horvat O, Jakovljevic V, Sabo J, Vasic R (2016). Erratum to: interaction of alcoholic extracts of hops with pentobarbital and diazepam in mice. European Journal of Drug Metabolism and Pharmacokinetics.

[ref-204] Ritz R, Scheidle C, Noell S, Roser F, Schenk M, Dietz K, Strauss WS (2012). *In vitro* comparison of hypericin and 5-aminolevulinic acid-derived protoporphyrin IX for photodynamic inactivation of medulloblastoma cells. PLOS ONE.

[ref-205] Roache JD, Griffiths RR (1987). Interactions of diazepam and caffeine: behavioral and subjective dose effects in humans. Pharmacology Biochemistry and Behavior.

[ref-206] Rodrigues AD, Lai Y, Shen H, Varma MVS, Rowland A, Oswald S (2019). Induction of human intestinal and hepatic organic anion transporting polypeptides: where is the evidence for its relevance in drug-drug interactions?. Drug Metabolism & Disposition.

[ref-207] Sacher J, Houle S, Parkes J, Rusjan P, Sagrati S, Wilson AA, Meyer JH (2011). Monoamine oxidase A inhibitor occupancy during treatment of major depressive episodes with moclobemide or St. John’s wort: an [11C]-harmine PET study. Journal of Psychiatry & Neuroscience.

[ref-208] Sansone RA, Sansone LA (2009). Tramadol: seizures, serotonin syndrome, and coadministered antidepressants. Psychiatry Journal.

[ref-209] Savage K, Firth J, Stough C, Sarris J (2018). GABA-modulating phytomedicines for anxiety: a systematic review of preclinical and clinical evidence. Phytotherapy Research.

[ref-210] Sawynok J (2011). Methylxanthines and pain.

[ref-211] Schempp CM, Lüdtke R, Winghofer B, Simon JC (2000). Effect of topical application of Hypericum perforatum extract (St. John’s wort) on skin sensitivity to solar simulated radiation. Photodermatology, Photoimmunology & Photomedicine.

[ref-212] Schempp CM, Müller K, Winghofer B, Schulte-Mönting J, Simon JC (2001). Single-dose and steady-state administration of Hypericum perforatum extract (St John’s Wort) does not influence skin sensitivity to UV radiation, visible light, and solar-simulated radiation. Archives of Dermatology Erratum.

[ref-213] Schempp CM, Winghofer B, Müller K, Schulte-Mönting J, Mannel M, Schöpf E, Simon JC (2003). Effect of oral administration of Hypericum perforatum extract (St. John’s wort) on skin erythema and pigmentation induced by UVB, UVA, visible light and solar simulated radiation. Phytotherapy Research.

[ref-214] Schneider-Yin X, Kurmanaviciene A, Roth M, Roos M, Fedier A, Minder EI, Walt H (2009). Hypericin and 5-aminolevulinic acid-induced protoporphyrin IX induce enhanced phototoxicity in human endometrial cancer cells with non-coherent white light. Photodiagnosis and Photodynamic Therapy.

[ref-215] Schumacher B, Scholle S, Hölzl J, Khudeir N, Hess S, Müller CE (2002). Lignans isolated from valerian: identification and characterization of a new olivil derivative with partial agonistic activity at A(1) adenosine receptors. Journal of Natural Products.

[ref-216] Sichardt K, Vissiennon Z, Koetter U, Brattström A, Nieber K (2007). Modulation of postsynaptic potentials in rat cortical neurons by valerian extracts macerated with different alcohols: involvement of adenosine A(1)- and GABA(A)-receptors. Phytotherapy Research.

[ref-217] Singh RK, Dillon B, Tatum DA, van Poppel KC, Bonthius DJ (2020). Drug-drug interactions between cannabidiol and lithium. Child Neurology Open.

[ref-218] Singh A, Zhao K (2017). Herb-drug interactions of commonly used Chinese medicinal herbs. International Review of Neurobiology.

[ref-219] Skalkos D, Gioti E, Stalikas CD, Meyer H, Papazoglou TG, Filippidis G (2006). Photophysical properties of *Hypericum perforatum* L. extracts—novel photosensitizers for PDT. Journal of Photochemistry and Photobiology B.

[ref-220] Skandhan AK, Ramakrishnan KG, Anand R (2013). Call-Fleming syndrome. Indian Journal of Radiology and Imaging.

[ref-221] Sklenář Z (2021). Magistraliter receptura v dermatologii.

[ref-222] Smith P, Bullock JM, Booker BM, Haas CE, Berenson CS, Jusko WJ (2004). The influence of St. John’s wort on the pharmacokinetics and protein binding of imatinib mesylate. Pharmacotherapy.

[ref-223] Smith PF, Maclennan K, Darlington CL (1996). The neuroprotective properties of the Ginkgo biloba leaf: a review of the possible relationship to platelet-activating factor (PAF). Journal of Ethnopharmacology.

[ref-224] Solimini R, Rotolo MC, Pellegrini M, Minutillo A, Pacifici R, Busardò FP, Zaami S (2017). Adulteration practices of psychoactive illicit drugs: an updated review. Current Pharmaceutical Biotechnology.

[ref-225] Spadaro A, Scott KR, Koyfman A, Long B (2022). High risk and low prevalence diseases: serotonin syndrome. The American Journal of Emergency Medicine.

[ref-226] Spinella M, Eaton LA (2002). Hypomania induced by herbal and pharmaceutical psychotropic medicines following mild traumatic brain injury. Brain Injury.

[ref-227] Stasiłowicz A, Tomala A, Podolak I, Cielecka-Piontek J (2021). *Cannabis sativa* L. as a natural drug meeting the criteria of a multitarget approach to treatment. International Journal of Molecular Sciences.

[ref-228] Stoddard GJ, Archer M, Shane-McWhorter L, Bray BE, Redd DF, Proulx J, Zeng-Treitler Q (2015). *Ginkgo* and warfarin interaction in a large veterans administration population.

[ref-229] Striley CW, Khan SR (2014). Review of the energy drink literature from 2013: findings continue to support most risk from mixing with alcohol. Current Opinion in Psychiatry.

[ref-230] Surendran S, Qassadi F, Surendran G, Lilley D, Heinrich M (2021). Myrcene—what are the potential health benefits of this flavouring and aroma agent?. Frontiers in Nutrition.

[ref-231] Svízenská I, Dubový P, Sulcová AP (2008). Cannabinoid receptors 1 and 2 (CB1 and CB2), their distribution, ligands and functional involvement in nervous system structures—a short review. Pharmacology Biochemistry & Behavior.

[ref-232] Tan KW, Cooney J, Jensen D, Li Y, Paxton JW, Birch NP, Scheepens A (2014). Hop-derived prenylflavonoids are substrates and inhibitors of the efflux transporter breast cancer resistance protein (BCRP/ABCG2). Molecular Nutrition & Food Research.

[ref-233] Tannergren C, Engman H, Knutson L, Hedeland M, Bondesson U, Lennernäs H (2004). St John’s wort decreases the bioavailability of R- and S-verapamil through induction of the first-pass metabolism. Clinical Pharmacology & Therapeutics.

[ref-234] Teufel-Mayer R, Gleitz JE (1997). Effects of long-term administration of *Hypericum* extracts on the affinity and density of the central serotonergic 5-HT1 A and 5-HT2 A receptors. Pharmacopsychiatry.

[ref-261] The World Flora Online (2022). *Thea sinensis* L. http://www.worldfloraonline.org/taxon/wfo-0000459564.

[ref-235] Tian J, Zhang F, Cheng J, Guo S, Liu P, Wang H (2014). Antidepressant-like activity of adhyperforin, a novel constituent of *Hypericum perforatum* L. Scientific Reports.

[ref-236] TRC (2023). Natural medicines.

[ref-237] Trépanier EF, Nafziger AN, Amsden GW (1998). Effect of terbinafine on theophylline pharmacokinetics in healthy volunteers. Antimicrobial Agents and Chemotherapy.

[ref-238] Tu P, Gibon J, Bouron A (2010). The TRPC6 channel activator hyperforin induces the release of zinc and calcium from mitochondria. Journal of Neurochemistry.

[ref-239] Tucker JS, Pedersen ER, Seelam R, Dunbar MS, Shih RA, D’Amico EJ (2019). Types of cannabis and tobacco/nicotine co-use and associated outcomes in young adulthood. Psychology of Addictive Behaviors.

[ref-240] Twardowski MA, Link MM, Twardowski NM (2019). Effects of cannabis use on sedation requirements for endoscopic procedures. Journal of Osteopathic Medicine.

[ref-253] Vázquez M, Guevara N, Maldonado C, Guido PC, Schaiquevich P (2020). Potential pharmacokinetic drug-drug interactions between cannabinoids and drugs used for chronic pain. BioMed Research International.

[ref-241] van Amerongen G, Siebenga P, de Kam ML, Hay JL, Groeneveld GJ (2018). Effect profile of paracetamol, Δ9-THC and promethazine using an evoked pain test battery in healthy subjects. European Journal of Pain.

[ref-242] van Beek TA (2005). Ginkgolides and bilobalide: their physical, chromatographic and spectroscopic properties. Bioorganic & Medicinal Chemistry.

[ref-243] van Beek TA (2000). Ginkgo biloba.

[ref-244] van Beek TA, Lelyveld GP (1992). Concentration of ginkgolides and bilobalide in *Ginkgo biloba* leaves in relation to the time of year. Planta Medica.

[ref-245] van Beek TA, Montoro P (2009). Chemical analysis and quality control of *Ginkgo biloba* leaves, extracts, and phytopharmaceuticals. Journal Chromatography A.

[ref-246] van Beek TA, Wintermans MS (2001). Preparative isolation and dual column high-performance liquid chromatography of ginkgolic acids from *Ginkgo biloba*. Journal of Chromatography A.

[ref-247] van Breemen RB, Chen L, Tonsing-Carter A, Banuvar S, Barengolts E, Viana M, Chen SN, Pauli GF, Bolton JL (2020). Pharmacokinetic interactions of a hop dietary supplement with drug metabolism in perimenopausal and postmenopausal women. Journal of Agricultural and Food Chemistry.

[ref-248] van Kan HJ, Groeneveld GJ, Kalmijn S, Spieksma M, van den Berg LH, Guchelaar HJ (2005). Association between CYP1A2 activity and riluzole clearance in patients with amyotrophic lateral sclerosis. British Journal of Clinical Pharmacology.

[ref-249] Van Soeren M, Mohr T, Kjaer M, Graham TE (1996). Acute effects of caffeine ingestion at rest in humans with impaired epinephrine responses. Journal of Applied Physiology.

[ref-250] Vaughn SE, Strawn JR, Poweleit EA, Sarangdhar M, Ramsey LB (2021). The impact of marijuana on antidepressant treatment in adolescents: clinical and pharmacologic considerations. Journal of Personalized Medicine.

[ref-251] Volpi-Abadie J, Kaye AM, Kaye AD (2013). Serotonin syndrome. Ochsner Journal.

[ref-252] Vulfsons S, Ognitz M, Bar-Sela G, Raz-Pasteur A, Eisenberg E (2020). Cannabis treatment in hospitalized patients using the SYQE inhaler: results of a pilot open-label study. Palliative & Supportive Care.

[ref-254] Walsh JK, Muehlbach MJ, Schweitzer PK (1995). Hypnotics and caffeine as countermeasures for shiftwork-related sleepiness and sleep disturbance. Journal of Sleep Research.

[ref-255] Wang Z, Hamman MA, Huang SM, Lesko LJ, Hall SD (2002). Effect of St John’s wort on the pharmacokinetics of fexofenadine. Clinical Pharmacology & Therapeutics.

[ref-256] Wang ZJ, Heinbockel T (2018). Essential oils and their constituents targeting the GABAergic system and sodium channels as treatment of neurological diseases. Molecules.

[ref-257] Wang Q, Zhao WZ, Ma CG (2000). Protective effects of Ginkgo biloba extract on gastric mucosa. Acta Pharmacologica Sinica.

[ref-258] Wanwimolruk S, Prachayasittikul V (2014). Cytochrome P450 enzyme mediated herbal drug interactions (Part 1). EXCLI Journal.

[ref-259] Wasowski C, Marder M (2012). Flavonoids as GABAA receptor ligands: the whole story?. Journal of Experimental Pharmacology.

[ref-260] Wesołowska O, Wiśniewski J, Sroda K, Krawczenko A, Bielawska-Pohl A, Paprocka M, Duś D, Michalak K (2010). 8-Prenylnaringenin is an inhibitor of multidrug resistance-associated transporters, P-glycoprotein and MRP1. European Journal of Pharmacology.

[ref-263] Williamson E, Driver S, Baxter K (2009). Stockley’s herbal medicines interactions.

[ref-264] Williamson E, Driver S, Baxter K (2013). Stockley’s herbal medicines interactions: a guide to the interactions of herbal medicines.

[ref-265] Williamson E, Driver S, Baxter K (2018). Stockley’s Phytopharmaka Interaktionen: Wechselwirkungen Pflanzlicher Arzneimittel.

[ref-266] Witte S, Anadere I, Walitza E (1992). Verminderung eines kardiovaskulären Risikofaktors [Improvement of hemorheology with *Ginkgo biloba* extract. Decreasing a cardiovascular risk factor]. Fortschritte der Medizin.

[ref-262] World Health Organization (2023). ATC code. www.whocc.no/atc.

[ref-267] Woziwodzka A, Krychowiak-Maśnicka M, Gołuński G, Łosiewska A, Borowik A, Wyrzykowski D, Piosik J (2022). New life of an old drug: caffeine as a modulator of antibacterial activity of commonly used antibiotics. Pharmaceuticals (Basel).

[ref-268] Yoshimaru T, Komatsu M, Tashiro E, Imoto M, Osada H, Miyoshi Y, Honda J, Sasa M, Katagiri T (2014). Xanthohumol suppresses oestrogen-signalling in breast cancer through the inhibition of BIG3-PHB2 interactions. Scientific Reports.

[ref-269] Yuan Y, Qiu X, Nikolić D, Chen SN, Huang K, Li G, Pauli GF, van Breemen RB (2014). Inhibition of human cytochrome P450 enzymes by hops (Humulus lupulus) and hop prenylphenols. European Journal of Pharmaceutical Sciences.

[ref-270] Zanoli P (2004). Role of hyperforin in the pharmacological activities of St. John’s wort. CNS Drug Reviews.

[ref-271] Zhang WY (2001). A benefit-risk assessment of caffeine as an analgesic adjuvant. Drug Safety.

[ref-272] Zhang Y, Man IC, Lai YS, Zuo Z (2022). Overview of current herb-drug interaction databases. Drug Metabolism & Disposition.

[ref-273] Zhang XY, Zhou DF, Su JM, Zhang PY (2001). The effect of extract of Ginkgo biloba added to haloperidol on superoxide dismutase in inpatients with chronic schizophrenia. Journal of Clinical Psychopharmacology.

[ref-274] Zhao M, Ma J, Li M, Zhang Y, Jiang B, Zhao X, Huai C, Shen L, Zhang N, He L, Qin S (2021). Cytochrome P450 enzymes and drug metabolism in humans. International Journal of Molecular Sciences.

[ref-275] Zhou S, Gao Y, Jiang W, Huang M, Xu A, Paxton JW (2003). Interactions of herbs with cytochrome P450. Drug Metabolism Reviews.

[ref-276] Ziemann J, Lendeckel A, Müller S, Horneber M, Ritter CA (2019). Herb-drug interactions: a novel algorithm-assisted information system for pharmacokinetic drug interactions with herbal supplements in cancer treatment. European Journal of Clinical Pharmacology.

[ref-277] Zuo XC, Zhang BK, Jia SJ, Liu SK, Zhou LY, Li J, Zhang J, Dai LL, Chen BM, Yang GP, Yuan H (2010). Effects of Ginkgo biloba extracts on diazepam metabolism: a pharmacokinetic study in healthy Chinese male subjects. European Journal of Clinical Pharmacology.

